# Microbial insights of enhanced anaerobic conversion of syngas into volatile fatty acids by co-fermentation with carbohydrate-rich synthetic wastewater

**DOI:** 10.1186/s13068-020-01694-z

**Published:** 2020-03-16

**Authors:** Chao Liu, Wen Wang, Sompong O-Thong, Ziyi Yang, Shicheng Zhang, Guangqing Liu, Gang Luo

**Affiliations:** 1 Biomass Energy and Environmental Engineering Research Center, Beijing University of Chemical Technology, Beijing, 100029 China; 2 Shanghai Key Laboratory of Atmospheric Particle Pollution and Prevention (LAP3), Department of Environment Science and Engineering, Fudan University, Shanghai, 200433 China; 3 Department of Biology, Faculty of Science, Thaksin University, Phathalung, 93110 Thailand; 4 Shanghai Institute of Pollution Control and Ecological Security, Shanghai, 200092 China

**Keywords:** Volatile fatty acid, Co-fermentation, Acetogen, qPCR analysis, Label-free quantitative proteomic analysis

## Abstract

**Background:**

The co-fermentation of syngas (mainly CO, H_2_ and CO_2_) and different concentrations of carbohydrate/protein synthetic wastewater to produce volatile fatty acids (VFAs) was conducted in the present study.

**Results:**

It was found that co-fermentation of syngas with carbohydrate-rich synthetic wastewater could enhance the conversion efficiency of syngas and the most efficient conversion of syngas was obtained by co-fermentation of syngas with 5 g/L glucose, which resulted in 25% and 43% increased conversion efficiencies of CO and H_2_, compared to syngas alone. The protein-rich synthetic wastewater as co-substrate, however, had inhibition on syngas conversion due to the presence of high concentration of NH_4_^+^-N (> 900 mg/L) produced from protein degradation. qPCR analysis found higher concentration of acetogens, which could use CO and H_2_, was present in syngas and glucose co-fermentation system, compared to glucose solo-fermentation or syngas solo-fermentation. In addition, the known acetogen *Clostridium formicoaceticum*, which could utilize both carbohydrate and CO/H_2_ was enriched in syngas solo-fermentation and syngas with glucose co-fermentation. In addition, butyrate was detected in syngas and glucose co-fermentation system, compared to glucose solo-fermentation. The detected *n*-butyrate could be converted from acetate and lactate/ethanol which produced from glucose in syngas and glucose co-fermentation system supported by label-free quantitative proteomic analysis.

**Conclusions:**

These results demonstrated that the co-fermentation with syngas and carbohydrate-rich wastewater could be a promising technology to increase the conversion of syngas to VFAs. In addition, the syngas and glucose co-fermentation system could change the degradation pathway of glucose in co-fermentation and produce fatty acids with longer carbon chain supported by microbial community and label-free quantitative proteomic analysis. The above results are innovative and lead to achieve effective conversion of syngas into VFAs/longer chain fatty acids, which would for sure have a great interest for the scientific and engineering community. Furthermore, the present study also used the combination of high-throughput sequencing of 16S rRNA genes, qPCR analysis and label-free quantitative proteomic analysis to provide deep insights of the co-fermentation process from the taxonomic and proteomic aspects, which should be applied for future studies relating with anaerobic fermentation.

## Background

Gasification or pyrolysis of lignocellulosic wastes produces a gas mixture (syngas), which is attracting much attention for the efficient conversion of the refractory organic wastes [1]. The produced syngas is mainly composed of CO, H_2_, CO_2_ (70–80%) and other trace compounds [2, 3]. The low volumetric energy density of syngas (usually less than 13 MJ/m^3^), which is only about 40% of natural gas, is the limitation for syngas to be used as fuel directly [3]. Alternatively, further conversion of syngas to more valuable biochemicals and biofuels is preferable, which can be metabolized by various microorganisms in anaerobic digestion [4, 5]. In recent years, many studies have been conducted to convert syngas to methane by anaerobic digestion (AD) [6, 7]. Compared with methane, the storage and transportation of volatile fatty acid (VFA) are easier and safer. In addition, it is estimated that the value of VFA from 1t biomass (150$) is much higher than that of methane (31$) [8]. Hence, anaerobic fermentation of syngas to produce VFA has attracted growing interests which can be used as an excellent carbon source for biological nutrients removal of wastewater directly, or as precursor to synthesize complex polymers such as PHA and longer chain fatty acids which are hydrophobic [9, 10]. The conversion of syngas to VFA by pure cultures was studied previously and *Clostridium* spp. (e.g., *Clostridium ljungdahlii*, *Clostridium carboxidivorans*, etc.) are the model microorganisms for VFA/ethanol production converted from syngas via Wood–Ljungdahl biochemical pathway [4, 11]. However, mixed culture is more promising compared with pure culture, considering no sterilization requirements, more adaptive capacity in various conditions due to microbial diversity and the possibility of using wastewater as nutrients in mixed culture fermentation. Previous studies also showed that H_2_ and CO can be converted to VFA metabolized by homoacegenesis and acetate was the main product using anaerobic mixed sludge [12, 13].

In recent years, researches on the VFA production by anaerobic fermentation of various organic wastes (such as food waste, wastewater) attract considerable attentions [14, 15], since it is the simple process for the simultaneous conversion of organic wastes and syngas to VFA. The VFAs/ethanol production from syngas and organic wastes has been performed using anaerobic fermentation, while which was mainly achieved by pure cultures [16, 17]. Although our previous study proposed a novel process for VFA production from integration process with syngas and waste activated sludge (WAS) [18], it was still unknown how the organic matters affect the production of VFA from syngas in the mixed culture. On the one hand, the conversion of syngas might be inhibited due to high concentrations of products generated from the anaerobic fermentation of organic wastes. Previous studies reported that high concentration of VFAs could inhibit bacterial growth during the anaerobic fermentation of syngas due to the presence of high concentrations of undissociated acids [4, 19]. On the other hand, the growth of some anaerobic bacteria such as acetogens can be accelerated by the presence of organic wastes which could also utilize CO and H_2_/CO_2_ by autotrophic pathway [4, 20]. Acevedo et al. compared the growth of *Clostridium ljungdahlii* between the syngas–fructose reactors and syngas reactors, and showed that the cell densities of *C. ljungdahlii* in syngas–fructose reactors were approximately 2.5 times higher than that in syngas reactors [16].

It is known protein and carbohydrate are the two main organic compounds in organic waste/wastewater, e.g., WAS and cassava stillage are the model protein-rich and carbohydrate-rich organic wastes which consisted of 65% protein and 60% carbohydrate, respectively [21, 22]. On the basis of the above considerations, the main objective of this work was to investigate the process performances of co-fermentation of syngas with carbohydrate or protein synthetic wastewater for VFA production, and to reveal the effects of different organic compounds on syngas fermentation. Label-free quantitative proteomic analysis was used to provide deep insights on the co-fermentation process from a microbiological point of view.

## Results

### Performances of the continuous bottles

#### Syngas fermentation with carbohydrate-rich synthetic wastewater for VFA production

**Fig. 1:**
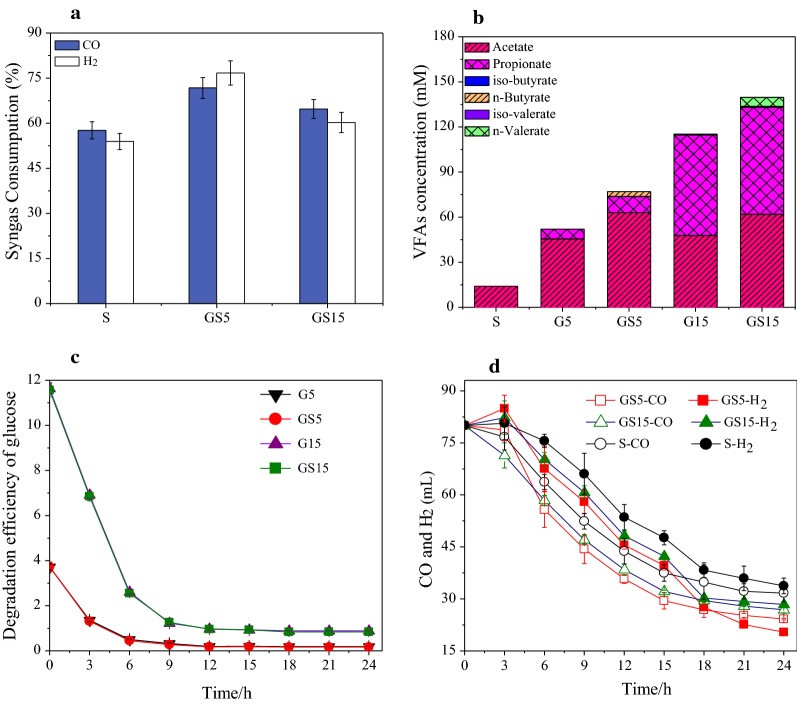
Performance in syngas–glucose co-fermentation bottles: **a** syngas consumption at steady state, **b** produced VFA in the effluent at steady state, **c** degradation of glucose in 24 h and **d** syngas consumption rate in 24 h. The steady state was defined by a sustained VFAs production and syngas consumption within ± 5% deviation. The CO/H_2_ conversion efficiencies calculated with the CO/H_2_ consumptions dividing by the total volume of CO/H_2_ in bottles. Ethanol production was insignificant (< 10 mg/L), and the concentration of ethanol was not shown in this study

**Fig. 2:**
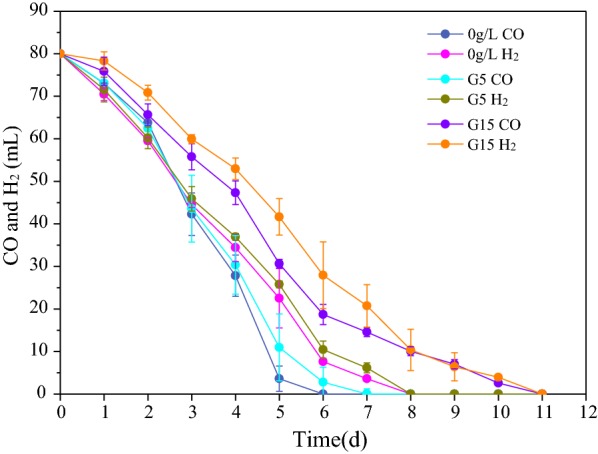
The consumption of syngas at different concentration of acetate and propionate which determined by the products concentration produced from glucose. G5: 45 mM acetate and 6 mM propionate; G15: 48 mM acetate and 67 mM propionate

As shown in Fig. 1a and Additional file 1: Fig. S2, the CO and H_2_ conversion efficiencies in S were 57% and 53%, respectively, while the conversion efficiencies of CO and H_2_ in GS5 were 25% and 43% (*P* < 0.05) higher compared to S. In the present study, no methane was detected, because 2-BES was added to the reactors at the beginning of the operation process. The time curves of CO and H_2_ consumption during 24 h in GS5 (*P*-value = 0.001, *F* = 28.3 > *F*_crit_ = 5.6 for CO; *P*-value = 0.026, *F* = 7.9 > *F*_crit_ = 5.5 for H_2_) were also higher than that in S (Fig. 1d). It was obvious that the presence of glucose in GS5 significantly enhanced the syngas conversion efficiencies (Additional file 1: Table S1). It was worthy to note that the syngas conversion efficiencies in GS15 was approximately 15% lower (*P* < 0.05) than that in GS5, which could be due to the negative effects of high concentrations of VFA (77.0 mM in GS5 vs. 139.7 mM in GS15). Previous studies showed that high VFA concentrations could have inhibition on the fermentation of organic compounds and syngas [19, 23]. The experiment about effect of acetate and propionate concentrations determined by the products concentration produced from glucose on syngas consumption was conducted (described in Additional file 1) and results showed that the acetate and propionate concentrations in G15 could inhibit the syngas conversion shown in Fig. 2. Nevertheless, the presence of glucose still promoted syngas conversion in GS15, further demonstrating the superiority for anaerobic co-fermentation of syngas and glucose.

**Fig. 3:**
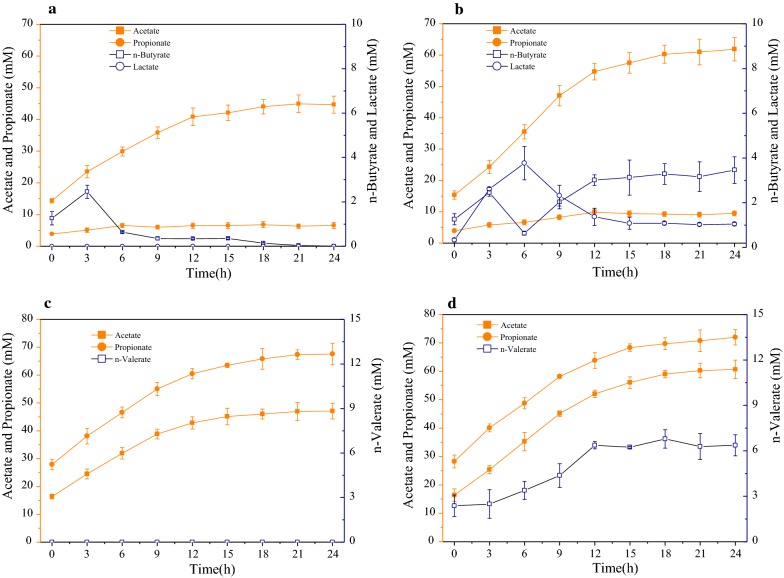
Time curves of VFAs production during 24 h for G5 (**a**), GS5 (**b**), G15 (**c**) and GS15 (**d**)

As shown in Fig. 1b and Table 1, only acetate (14.1 mM) was detected in S by the conversion of syngas and the acetate conversion efficiency from syngas was 96%. The concentrations of acetate, propionate and *n*-butyrate were all increased in GS5 compared to G5. The concentration of acetate in GS5 was 38% higher than that in G5 (62.9 Mm vs. 45.6 mM, *P* < 0.05) which could be mainly due to the conversion of syngas. Higher concentrations of propionate and *n*-butyrate were obtained in GS5 (10.9 mM in GS5 vs. 6.4 mM in G5, 3.2 mM in GS5 vs. 0 mM in G5, *P* < 0.05), and 1.2 mM lactate was detected only in the effluent of GS5. The time curves of VFA concentrations after feeding (24 h) during the steady-state were measured to determine the metabolism pathway in the co-fermentation system of syngas and glucose. As shown in Fig. 3a, b, acetate and propionate were the final products in both G5 and GS5. However, lactate was only detected in GS5 as an intermediate, which indicated that the co-fermentation of syngas with glucose could result in the change of glucose degradation pathway. Obvious decrease of lactate concentration and increase of *n*-butyrate concentration were detected after 6 h’ fermentation. Therefore, it seems *n*-butyrate was converted from lactate.

**Table 1:** Summary of the experimental performances

	G5	GS5	G15	GS15	P5	PS5	P15	PS15	S
CO consumption (mL/day)	–	57.4 ± 2.8	–	51.8 ± 2.5	–	26.1 ± 1.4	–	21.9 ± 2.3	45.6 ± 2.1
H_2_ consumption (mL/day)	–	61.4 ± 3.2	–	48.2 ± 2.7	–	27.5 ± 2.2	–	21.1 ± 2.7	42.4 ± 2.8
CO conversion efficiency (%)^a^	–	71.7 ± 3.5	–	64.7 ± 4.0	–	32.6 ± 1.8	–	27.3 ± 2.9	57.6 ± 2.8
H_2_ conversion efficiency (%)^a^	–	76.7 ± 1.2	–	60.3 ± 0.9	–	34.4 ± 0.7	–	26.4 ± 2.1	53.0 ± 1.7
Acetate conversion efficiency from syngas (%)^b^	–	90 ± 2		69 ± 1	–	153 ± 3	–	212 ± 5	96 ± 2
VFAs conversion efficiency from organic (%)^c^	84 ± 1	92 ± 4	81 ± 2	84 ± 2	95 ± 3	93 ± 2	94 ± 4	93 ± 5	–
Glucose/BSA (mg/L)	230 ± 19	227 ± 29	1256 ± 136	1194 ± 211	725 ± 37	794 ± 71	3584 ± 203	3346 ± 309	296 ± 19
Total COD (mg COD/L)^d^	5300	6822	15,900	17,422	7100	8622	21,300	22,822	1522
VFAs/total COD (%)^d^	82 ± 1.2	86 ± 2.3	75 ± 0.9	78 ± 2.5	82 ± 1.0	70 ± 2.1	64 ± 2.7	63 ± 3.2	55 ± 1.3
Residual syngas/total COD (%)^d^	–	6 ± 0.5	–	4 ± 0.3	–	10 ± 0.8	–	5 ± 0.4	40 ± 1.2
Residual organic/total COD (%)^d^	5 ± 0.2	3 ± 0.1	9 ± 0.4	8 ± 0.7	11 ± 1.4	10 ± 0.8	24 ± 1.9	22 ± 2.2	–
Unbalanced COD/total COD (%)^d^	12 ± 2.5	4 ± 0.1	15 ± 2.2	11 ± 3.1	6 ± 0.5	9 ± 1.3	11 ± 1.6	10 ± 0.9	5 ± 1.3
NH_4_^+^-N (mg/L)	80 ± 5	87 ± 17	78 ± 4	80 ± 5	935 ± 46	916 ± 49	1608 ± 91	1623 ± 63	

^a^The *P*-values of CO/H_2_ conversion efficiency among all the reactors were calculated by Duncan analysis and shown in Additional file 1: Table S1

^b^One mole acetate was produced from 4 mol H_2_ or 4 mol CO (4CO + 2H_2_O → CH_3_COOH + 2CO_2_ and 4H_2_ + 2CO_2_ → CH_3_COOH + 2H_2_O), and the theoretical acetate production from H_2_ and CO was calculated according to the above two stoichiometric equations. The acetate conversion efficiency from converted CO and H_2_ was calculated: the measured acetate production/ the theoretical acetate production from H_2_ and CO

^c^The theoretical VFA production (mgCOD/L) from organic was calculated by subtracting the COD of residual organic from the COD of added organic. The VFAs conversion efficiency from organic was calculated: the measured VFAs production/the theoretical VFAs production from organic

^d^COD mass balance in all bottles at steady state. Total COD was the sum of organic and syngas. COD was calculated based on the following: CO: 0.57 gCOD/g, H_2_: 8 gCOD/g, ACETATE: 1.07 gCOD/g, propionate: 1.51 gCOD/g, butyrate: 1.81 g COD/g, valerate: 2.04 g COD/g, carbohydrate: 1.06 g COD/g, protein: 1.42 g COD/g, lactate: 1.06 gCOD/g. Unbalanced COD was calculated by subtracting the COD of VFAs, residual syngas, residual organic from total COD

Different from G5 and GS5, both propionate and acetate were the main fermentation products in G15 and GS15, which could be mainly due to the higher glucose concentration in G15 and GS15. Previous studies also reported that the concentration of substrate affects the final products [24, 25]. Obviously higher concentrations of acetate and *n*-valerate were detected in GS15 compared to G15. In GS15, the acetate conversion efficiency from syngas was only 69% (Table 1), which indicated that around 30% of the consumed syngas was not converted to acetate but to other products. It was possible that high concentration of propionate compared with acetate resulted in *n*-valerate production by propionate with syngas in GS15, but not in GS5 [26, 27]. As shown in Table 1, there was no significant difference in the glucose concentrations in the effluent of G5 and GS5 as well as G15 and GS15. In addition, the glucose degradation rates were also not obviously changed in syngas and glucose co-fermentation system (Fig. 1c) by measuring the time curves of glucose concentrations in bottles after feeding for one cycle during the steady-state. The above results indicated that the co-fermentation of syngas with glucose did not have negative effect on glucose conversion.

#### Syngas fermentation with protein-rich synthetic wastewater for VFA production

**Fig. 4:**
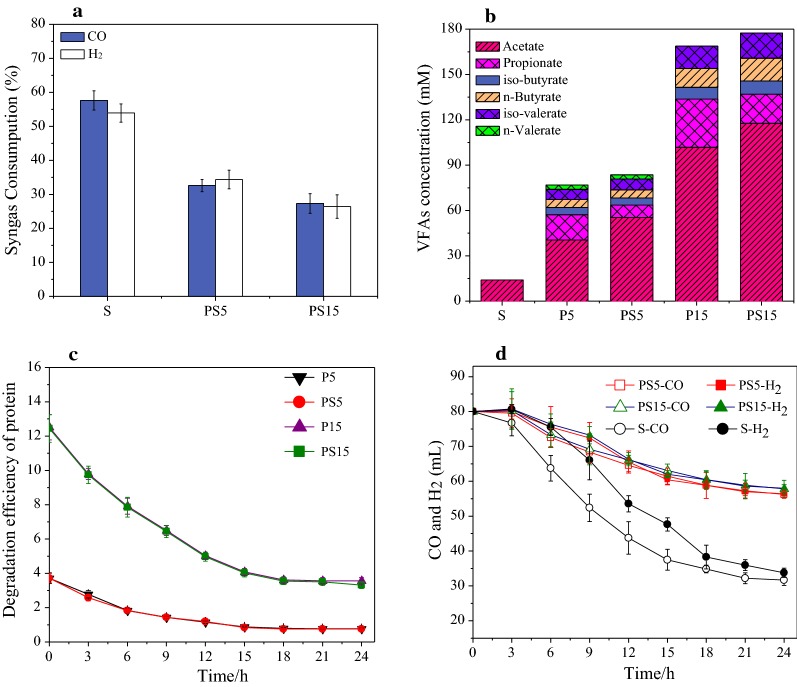
Performance in syngas–BSA co-fermentation bottles: **a** syngas consumption at steady-state, **b** produced VFA in the effluent at steady state, **c** degradation of BSA in 24 h and **d** syngas consumption rate in 24 h

As shown in Fig. 4a, the consumption efficiencies of CO and H_2_ were 32% and 34% in PS5, and they were 27% and 26% in PS15, which were significantly lower (43–52% for CO, 35–51% for H_2_, *P* < 0.05) than that in S (57% for CO and 53% for H_2_). The time curves of CO and H_2_ consumption during 24 h in PS5 (*P*-value = 0.004, *F* = 8.0 > *F*_crit_ = 3.4 for CO; *P*-value = 0.002, *F* = 9.1 > *F*_crit_ = 3.5 for H_2_) were also higher than that in S (Fig. 4d). The above results indicated the presence of BSA during anaerobic fermentation have inhibition on the conversion of syngas. There might be some products during BSA degradation inhibiting the syngas fermentation. VFA are the main products for anaerobic fermentation of protein, however, their concentrations in the present study, especially PS5 (around 90 mM), was not high enough to have inhibition since it achieved around 130 mM in GS15 without inhibiting syngas fermentation. NH_4_^+^-N was also a product from the fermentation of BSA. Previous studies demonstrated that there were inhibitory effects on the biogas production in AD reactor when the NH_4_^+^-N was present especially at alkaline pH [28, 29]. As shown in Table 1, the concentrations of NH_4_^+^-N (> 900 mg/L) were much higher in bottles with BSA as co-substrate than that (< 100 mg/L) in bottles with glucose as co-substrate. Therefore, it was possible the presence of high concentration of NH_4_^+^-N had inhibition on syngas fermentation. Further experiments were also carried out to investigate the effect of NH_4_^+^-N concentration on the conversion of syngas. As shown in Additional file 1: Fig. S3, CO could be consumed completely in 4 days, and H_2_ could also be consumed completely in 6 days in the bottle with NH_4_^+^-N concentration of 0 mg/L. However, there were still around 25% of the initial CO and H_2_ left after 8 days of fermentation in bottle with NH_4_^+^-N concentration of 1000 mg/L. It indicated that high NH_4_^+^-N concentration had inhibition on the consumption of syngas. Therefore, it might be not suitable for the co-fermentation of syngas and protein-rich organic wastes.

It should be noted that acetate was the main fermentation product in all BSA-bottles, and the concentration of acetate was increased in syngas with BSA co-fermentation system in Fig. 4b (55.4 mM in PS5 vs. 40.5 mM in P5, and 117.6 mM in PS15 vs. 101.9 mM in P15, *P* < 0.05). The time curves of BSA concentration in P5, PS5, P15 and PS15 are reported in Fig. 4c, where there is also no significant difference in the degradation rates of BSA in PS5 and PS15 compared to P5 and P15. Although the degradation rate of proteins was not affected in syngas and BSA co-fermentation system, it was not suitable for co-fermentation of syngas and protein-rich synthetic wastewater due to the inefficient conversion of syngas.

### Microbial community compositions analysis

To reveal the differences of microbial community compositions among the inoculum and 9 samples collected from the bottles, the richness and diversity of microbial community were studied using high-throughput sequencing of the 16S rRNA genes. The changes of the microbial community among the samples were revealed by the Rectangular cladogram which was based on Bray–Curtis similarity matrices [18]. From Additional file 1: Fig. S4A, it can be clearly seen that the clustering of inoculum and sample S, which was separated from the others. The samples obtained from the bottles with the same organic matters (glucose or BSA) were generally clustered together (G5 and G15, P5 and P15) indicating the different types of organics could change the microbial community compositions obviously during the anaerobic fermentation. In addition, the co-fermentation of syngas with organic matter obviously changed the microbial communities of glucose or BSA based bottles; e.g., samples GS5 and GS15 were clustered separately from G5 and G15. Principle component analysis (PCA) based on OTU analysis at 0.03 distance (97% sequence similarity of OTUs) (Additional file 1: Fig. S4B) also showed similar results. The above findings clearly suggested that both syngas and co-substrate types played important roles in shaping the microbial community compositions.

**Fig. 5:**
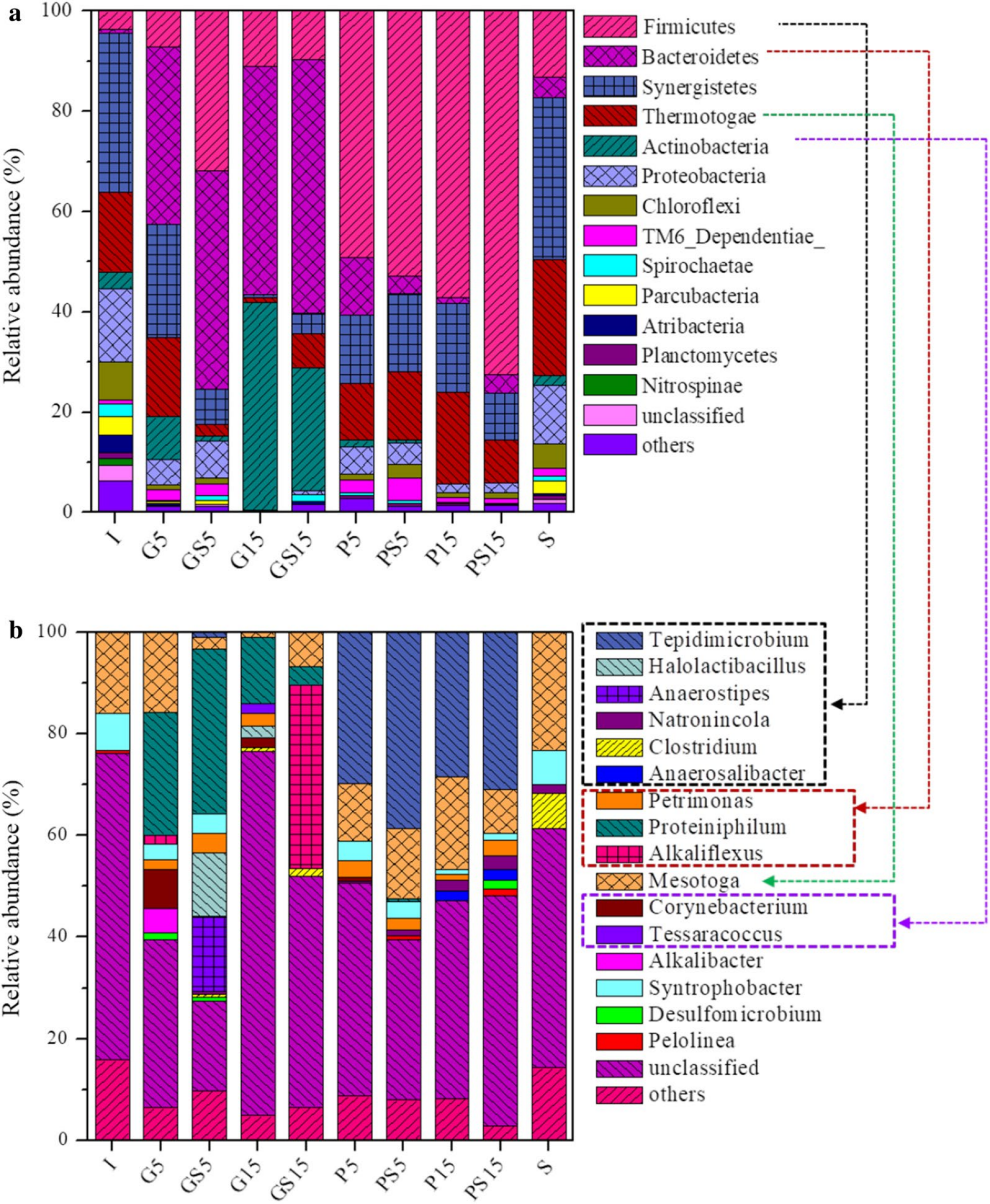
Taxonomic classification of the microbial community: comparison of the relative abundances at **a** phylum and **b** genus levels of each sample

The relative abundances of *Thermotogae* (23%), *Firmicutes* (13%) and *Bacteroidetes* (4%) in S were higher compared to that in inoculum (15.9%, 3% and 0.7%), indicating the enrichment of *Thermotogae*, *Firmicutes* and *Bacteroidetes* in S (Fig. 5a). The phylum *Bacteroidetes*, *Synergistetes* and *Themotogae* were the dominant microorganisms in G5, while *Bacteroidetes* and *Actinobacteria* were dominant in G15. A higher relative abundances of *Firmicutes* (31%) in GS5 was obtained compared to that in G5 (7%) and a higher relative abundances of *Thermotogae* (6%) was obtained in GS15 compared to that in G15 (1%). The relative abundance of *Firmicutes* was increased when the concentration of BSA increased from 5 to 15 g g/L (e.g., 49% in P5 and 57% in P15). In addition, higher relative abundances of *Firmicutes* (52 and 72%) were obtained in PS5 and PS15 compared with that in P5 and P15. The above results indicated that the concentration of organic matter and the co-fermentation of syngas with organic matter could significantly change the microbial community compositions.

The genus-level classification (Fig. 5b) showed that *Halolactibacillus* and *Anaerostipes* were obviously boosted in GS5 (GS5 12%, G5 1%). The genus *Alkaliflexus* was obviously enriched in GS15 (36% in GS15 vs. 1% in G15), but higher relative abundances of *Proteiniphilum* was obtained in G15 (15% in G15 vs. 4% in GS15), both of which were the known bacteria that could degrade glucose and produce propionate [30, 31]. The above results further indicated that the co-fermentation of syngas with glucose could shape the microbial community structure in the degradation of glucose. In addition, the known bacteria *Natronincola* and *Clostridium* that have the ability of CO/H_2_-utilizing were found in the present study [32, 33]. The genus *Tepidimicrobium* was obviously enriched in bottles with syngas; e.g., the relative abundances were 39 and 31% in PS5 and PS15, while they were 29 and 27% in P5 and P15.

Venn diagram based on 0.03 distance showed that the shared OTUs in bottles with and without syngas accounted for above 40% (Additional file 1: Fig. S5). OTU based analysis was conducted and the OTUs which were enriched in co-fermentation bottles were analyzed as shown in Additional file 1: Tables S2 and S4. The relative abundances of OTU3 and OTU4 were higher in S compared with that in I, and they had 90% similarity to the known acetogen (*Natronincola histidinovorans* and *Clostridium formicoaceticum*) [34, 35] which showed the enrichment of syngas-utilizing bacteria in S. In addition, *Natronincola histidinovorans* and *Clostridium formicoaceticum* were also enriched in co-fermentation with syngas and glucose. As shown in Additional file 1: Table S2, most of the other OTUs were similar with known bacteria (*Halolactibacillus alkaliphilus, Lascolabacillus massiliensis, Aminobacterium colombiense* and *Tepidimicrobium ferriphilum*) which grow chemoorganotrophically [36–38]. In addition, the OTUs analysis showed that OTU 2 enriched in PS5 and PS15 had high similarity to *Tepidimicrobium ferriphilum* (91%). The OTUs analysis also showed that *Natronincola histidinovorans* was enriched in PS5 and PS15 which could be related to syngas conversion.

### qPCR analysis

**Fig. 6:**
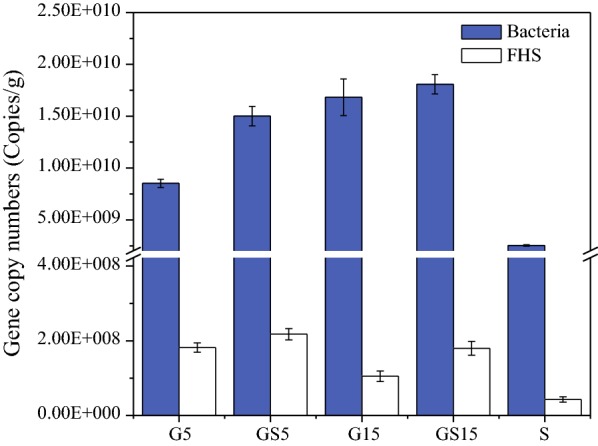
qPCR analysis of the selected genes in samples of inoculum and syngas–glucose co-fermentation bottles

The present study showed that glucose as co-substrate was more suitable for efficient syngas conversion for acetate production. In consequence, the concentrations of total bacteria and acetogens in G5, G15, GS5, GS15 and S were determined as revealed by qPCR analysis (Fig. 6). The higher concentrations of total bacteria were obtained in co-fermentation bottles by comparison to that in glucose solo-fermentation bottles, which could be due to the conversion of more substrates. The concentrations of acetogens were higher in GS5 and GS15 compared to G5 and G15. It was worth noting that the concentration of acetogens in G5 was much higher than that in S, indicating G5 had high potential to utilize syngas.

### Proteomic insights into the syngas and carbohydrate-rich co-fermentation system

**Fig. 7:**
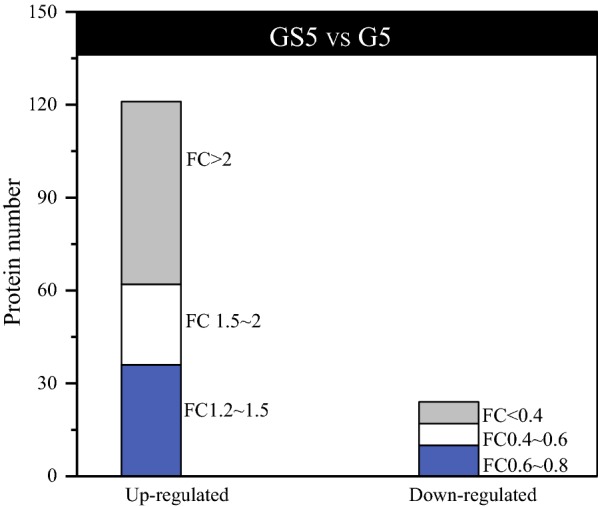
Numbers of up-regulated and down-regulated proteins in GS5 compared with G5

The VFA production from organic wastewater is attracting much attention recently, and the addition of syngas into the fermentation of glucose wastewater is a simple method for syngas fermentation. The present study showed that glucose metabolic pathways changed during co-fermentation with syngas, and therefore the underlying mechanism deserved to be elucidated. Label-free quantitative proteomic technology was performed to analyze the changes of protein expression levels and determine differentially expressed proteins (145) in GS5 compared to G5 since GS5 was most efficient for syngas conversion (Fig. 7). 121 proteins were highly up-regulated and 24 proteins were down-regulated in GS5 compared to G5.

The Gene Ontology (GO) terms and KEGG database were further analyzed to determine the category enrichment and metabolic pathways of the differentially expressed proteins in GS5 compared to G5 [39]. As shown in Additional file 1: Table S5, the carbon utilization, cellular component organization, biogenesis, binding and catalytic activity by GO enrichment analysis were significantly enriched (*P* < 0.05). As shown in Additional file 1: Table S6, KEGG database showed that the differentially expressed proteins were enriched in glycolysis/gluconeogenesis (13.7%), biosynthesis of amino acids (15.4%), carbon metabolism (37%), and biosynthesis of antibiotics (27.5%).

In addition, differentially expressed protein involving in enriched metabolic pathway was analyzed to reveal the mechanism of enhanced VFA production by syngas co-fermented with glucose in detail. Table 2 shows that the differentially expressed proteins, relating with key enzymes (e.g. carbon-monoxide dehydrogenase/CODH, acetyl-CoA synthase/ACS, 5-methyltetrahydrofolate, formyltetrahydrofolate synthetase) in the syngas metabolism were up-regulated. The key enzymes in the glucose fermentation affected by the co-fermentation of syngas with glucose are shown in Tables 3 and 4. Five differentially expressed proteins (2 up-regulated and 3 down-regulated) relating with pyruvate synthase were detected, which was a key enzyme in glucose metabolism [21]. Label-free quantitative proteomic technology showed that the electron transfer flavoprotein FAD-binding and phosphate butyryltransferase (PTB responsible for *n*-butyrate formation from butyrate-CoA) domain protein related proteins were also up-regulated, and the up-folds were 3.79 and 16, respectively. In the present study, higher concentration of propionate was obtained in GS5 compared to G5, and the differentially expressed proteins, relating with succinyl-CoA synthetase originated from *Syntrophobacter* in the propionate production were up-regulated, which were also detected by the high-throughput sequencing of 16S rRNA gene. Overall, the co-fermentation of syngas with glucose induced differentially expression level of key enzymes involved in glucose degradation, and the up-regulated expression of key enzymes involved in butyrate production.

**Table 2:** The expression level of differentially expressed proteins in acetate production from syngas in GS5 compared with G5

Name	Up-regulated (fold)	Taxonomy assignment
Carbon-monoxide dehydrogenase	A0A1D9FRD2 (16)	*Clostridium*
	A0A1G9EJN0 (16)	*Natronincola*
Acetyl-CoA synthase	A0A1D9FLX9 (8.79)	*Clostridium*
	A0A1D9FSJ8 (16)	*Clostridium*
5-Methyltetrahydrofolate	A0A1D9FMJ4 (16)	*Clostridium*
Formyltetrahydrofolate synthetase	I5AR69 (16)	*Eubacterium*
	A0A1M6LF53 (16)	*Paramaledivibacter*
	A0A1C0BIW9 (16)	*Clostridium*
	A0A1G5JRT7 (16)	*Alkaliphilus*
	A0A1D9FM79 (240)	*Clostridium*
	A0A1M5S461 (16)	*Caloranaerobacter*

**Table 3:** The expression level of differentially expressed proteins in glucose degradation in GS5 compared with G5

Name	Up-regulated (fold)	Taxonomy assignment	Down-regulated (fold)	Taxonomy assignment
3-Methyl-2-oxobutanoate dehydrogenase	R5V805 (16)	*Alistipes*		
			A0A0C3RE68 (0.0625)	*Sanguibacteroides*
			A0A0B2JG02 (0.0625)	*Coprobacter*
Pyruvate synthase	A0A1C6CVU3 (16)	*Eubacterium*		
	A0A1I6NQB3 (1.94)	*Porphyromonadaceae*		
			A0A1M6JBF2 (0.09)	*Lutispora*
			A0A1M4XQ81 (0.14)	*Alkalibacter*
			A0A1W9VKC5 (0.06)	*Anaerolineaceae*
Phosphoenol-transphosphorylase	A0A1C5XC36 (16)	*Eubacterium*		
Phosphoenolpyruvate-carboxykinas	A0A1I6PSE0 (1.31)	*Porphyromonadaceae*		
			A0A098C4G8 (0.0625)	*Fermentimonas*
Succinyl-CoA synthetase	A0A2U3L9L1 (12.34)	*Syntrophobacter*

**Table 4:** The expression level of differentially expressed proteins in butyrate production in GS5 compared with G5

Name	Up-regulated (fold)	Taxonomy assignment
Alcohol dehydrogenase	A0A1D9FIQ3 (8.99)	*Clostridium*
	T0PGH4 (16)	*Clostridium*
	A0A1M4XVX5 (65.518)	*Alkalibacter*
	A0A1C5SNP8 (16)	*Clostridium*
Phosphate butyryltransferase	B1CB75 (16)	*Anaerofustis*
	A0A1Q9JJJ6 (3.74)	*Eubacterium*
Electron transfer flavoprotein FAD-binding domain protein	B0M9M9 (3.79)	*Anaerostipes*

## Discussion

The present study showed that 5 g/L carbohydrate-rich synthetic wastewater was more suitable as co-substrate for fermentation of syngas since it could significantly enhance the syngas conversion (25% and 43% of increased conversion efficiencies of CO and H_2_, compared to syngas alone, *P* < 0.05) and VFA production. Previous studies showed that some anaerobic bacteria that could utilize glucose belong to acetogens, which could also utilize CO and H_2_/CO_2_ by autotrophic pathway [4, 5]. It is known that bacteria grow faster by heterotrophic pathway than autotrophic pathway. Therefore, the presence of glucose might promote the growth of bacteria, and thereby enhanced utilization of syngas. And the higher concentration of acetogens detected in GS5 compared to G5 and S in qPCR analysis supported that acetogens played important roles in the syngas conversion for acetate production. The co-fermentation of syngas with glucose in GS5 further promoted the growth of acetogens compared to glucose solo-fermentation in G5, and thereby higher syngas conversion rates were achieved in GS5 compared to S. In addition, the known acetogen *Clostridium formicoaceticum*, which is able to grow either on CO and H_2_/CO_2_ by autotrophic pathway, or grow on organic matter by Embden–Meyerhof–Parnas pathway and the pentose phosphate pathway, also showed that the growth of acetogens could be promoted in GS5. Although several acetogens besides *C*. *formicoaceticum* are known to be able to ferment both glucose and syngas (e.g., *C. autoethanogenum*, *C. carboxidivorans*, *C. ljungdahlii*, etc.) [4, 40], only *C*. *formicoaceticum* was enriched, which might be related with the suitable co-fermentation condition in the present study supported by the specific niches of each microorganism [33, 41]. Furthermore, label-free quantitative proteomic analysis showed that the up-regulated differentially expressed proteins (carbon-monoxide dehydrogenase and acetyl-CoA synthase), relating with key enzymes in the acetate production from syngas metabolism was up-regulated, which was consistent with the highest conversion of syngas and production of acetate in Fig. 1. Some of the above differentially expressed proteins were originated from known acetogens (e.g. *Clostridium*, *Natronincola*) [34, 35], which were also detected by the microbial community compositions analysis as mentioned before.

It is worth noting that *n*-butyrate was detected in GS5 compared to G5 which might be related with the change of glucose/syngas metabolism pathway due to the selection of bacteria by the co-fermentation of syngas with glucose and previous studies showed that butyrate could be produced from electron donor and acetate [42–44]. For examples, both H_2_ and CO had been reported to be as electron donors to upgrade acetate to *n*-butyrate [26, 40]. The acetate conversion efficiency from syngas in GS5 achieved 90%, which indicated that the consumed syngas was stoichiometrically converted to acetate (Table 1). Lactate was detected only in the effluent of GS5, and previous studies showed that lactate can also be used as electron donor to synthesis *n*-butyrate with acetate [42, 43] which could be supported by the time curves of VFA concentrations after feeding (24 h) during the steady state. The microbial community analysis showed that *Halolactibacillus alkaliphilus* could degrade glucose and the main product was lactate, which was consistent with the detection of lactate as intermediate in GS5 (Fig. 3b), and *Anaerostipes rhamnosivorans* could produce *n*-butyrate from acetate and lactate, which was also consistent with the detection of *n*-butyrate in GS5. It seems that the syngas co-fermentation with glucose changed the degradation pathway of glucose to produce *n*-butyrate via lactate as an intermediate. The key enzymes pyruvate synthase in the glucose fermentation were also affected in GS5, however, the comprehensive expression alteration of pyruvate synthase could not be evaluated considering that the differentially expressed proteins which were assigned to the same enzyme might derive from different microbes (52). Nevertheless, the changes and sourced microorganisms of each differentially expressed protein could be clarified by the label-free quantitative proteomic technology. It was clear that the up-regulated proteins related to pyruvate synthase were derived from the genus *Eubacterium* and *Porphyromonadaceae*, while the down-regulated proteins assigned to pyruvate synthase were derived from the genus *Lutispora*, *Alkalibacter*, and *Anaerolineaceae*. The above results suggested that the co-fermentation changed the expression of proteins which were assigned to pyruvate synthase from some bacteria selectively. As previously mentioned, *Anaerostipes* could produce *n*-butyrate from acetate and lactate [44], the proteins electron transfer flavoprotein FAD-binding domain protein related proteins from *Anaerostipes* was up-regulated, and it indicated that electron transfer flavoprotein FAD-binding domain protein was related with *n*-butyrate production. The PTB related protein was originated from *Eubacterium*, which could produce *n*-butyrate from acetate [10]. However, the *Eubacterium* had low abundance by the microbial community analysis, which might be due to their high activity. In addition, the differentially expressed protein relating alcohol dehydrogenase (ADH responsible for acetyl-CoA formation from ethanol) derived from *Clostridium* was up-regulated, which could produce *n*-butyrate from acetate and ethanol. However, ethanol was not detected in our study, which might be due to the rapid consumption of ethanol. Therefore, the glucose degradation pathway changed in the co-fermentation of syngas and glucose was confirmed in protein expression levels involved in various pathways.

However, it was not suitable for co-fermentation of syngas and BAS considering the inefficient conversion of syngas which was due to the high concentration of NH_4_^+^-N produced during BSA degradation. The measured increase of acetate concentration was significantly higher than the theoretical acetate production from consumed syngas, which could be due to the changes of degradation pathway of BSA. A previous study showed that Stickland reaction was the main metabolic pathway for degradation of amino acid when the pressure of H_2_ was high [45]. The metabolic pathway for degradation of amino acid via Stickland reaction was showed in Additional file 1: Fig. S7. The genus *Tepidimicrobium*, performing the Stickland reaction, was obviously enriched in PS5 and PS15 compared to that in P5 and P15, which was consistent with higher percentage of acetate obtained in PS5 and PS15. Previous studies indicated that the genus *Tepidimicrobium* is mainly composed by two species (*Tepidimicrobium xylanilyticum* and *Tepidimicrobium ferriphilum*) [46, 47]. And *Tepidimicrobium ferriphilum* as the representative strain could perform the Stickland Reaction. The above findings further suggested that the co-fermentation of syngas with BSA could affect the pathway for the metabolism of protein.

### Implication

The present study developed an efficient process to convert syngas to volatile fatty acids (VFAs) by co-fermenting with carbohydrate-rich synthetic wastewater since it could promote the growth of acetogens and also would not produce high-concentration VFAs. The protein-rich wastewater was not suitable for the co-fermentation with syngas due to the production of high concentration of NH_4_^+^-N (> 900 mg/L). It should be noted fatty acids with longer carbon chain (e.g., butyrate and valerate in Fig. 1b) were produced for the co-fermentation of syngas with carbohydrate-rich wastewater, and currently fatty acids with longer carbon chain has attracted much attention due to their low solubility and high energy content [4, 40]. Therefore, the co-fermentation process can also be optimized in order to produce longer chain fatty acids in the future. Most previous studies only focused on taxonomic analysis for anaerobic fermentation of syngas and organic wastes [18, 48], which could not elucidate the exact roles of the microorganisms. Label-free quantitative proteomic analysis helped to understand the metabolic pathways taking place during the fermentation process. The combination of high-throughput sequencing of 16S rRNA genes, qPCR analysis and label-free quantitative proteomic analysis used in the present study provided deep insights of the co-fermentation process for syngas and carbohydrate wastewater from the taxonomic and proteomic aspects, which should be applied for future studies relating with anaerobic fermentation. The present study was conducted to investigate the effect of different synthetic wastewater and different concentration on syngas conversion in lab-scale reactors, and more efforts are needed to accelerate its full-scale application.

## Conclusion

The present study developed a new process to increase the mixed culture conversion of syngas to VFA by co-fermenting with glucose synthetic wastewater, and the most efficient conversion of CO and H_2_ (71% and 76%, respectively) was obtained with 5 g/L carbohydrate wastewater as co-substrate, which could be related with the higher concentration of acetogens promoted by glucose in co-fermentation as proved by qPCR analysis. In contrast, the presence of protein inhibited syngas conversion efficiencies due to high concentration of NH_4_^+^-N produced by protein degradation. Further study deserved to be conducted to see whether the removal of NH_4_^+^-N from the fermentation process by various methods (stripping, emulsion liquid membrane, and electrochemical oxidation) could promote syngas conversion by co-fermentation with protein-rich wastewater. High-throughput sequencing analysis of 16S rRNA genes showed that some known CO/H_2_-utilizing acetogens were enriched in co-fermentation of syngas with glucose compared with glucose alone. And it was worthy to note that longer chain fatty acid (e.g., butyrate) was detected in syngas and glucose co-fermentation system, which could be converted by acetate and lactate/ethanol produced from glucose. Therefore, it is possible to produce more valuable longer chain fatty acid in the co-fermentation of syngas and carbohydrate wastewater. Label-free quantitative proteomic analysis gave further insights on the changes of protein expression levels involved in various pathways induced by the co-fermentation of syngas with glucose compared to glucose solo-fermentation. The up-regulation of proteins relating with key enzymes involved in acetate and butyrate production were found which revealed the metabolic pathways taking place during the co-fermentation process.

## Materials and methods

### Inoculum and feedstock

The inoculum was collected from a mesophilic anaerobic reactor treating cassava stillage in an ethanol plant. Glucose and bovine serum albumin (BSA) were used as the model compounds of carbohydrate and protein, respectively [49, 50]. They were dissolved in basic medium (BA medium) to prepare the feedstock solutions. In addition, the feedstock solutions also contained carbonate/bicarbonate buffer solution (50 mM and pH 9.0) in order to keep the pH as stable as possible during the fermentation. The composition of BA medium was described in previous study [51]. Syngas was composed of 40% H_2_; 40% CO; 20% CO_2_.

### Set-up of the experiments

Serum bottles (320 mL) were filled with 120 mL medium having different concentration of either glucose or BSA. Different concentrations of glucose (5 and 15 g/L) were used as the co-substrates in four bottles (G5, GS5, G15 and GS15). G5 and G15 were fed with only glucose, which were used as control bottles, and the other two bottles were fed with both glucose and syngas. Different concentrations of BSA (5 and 15 g/L) were used as the co-substrate in the other four bottles (P5, PS5, P15 and PS15). The control bottles (P5 and P15) were fed with only BSA, and both BSA and syngas were added to the bottles PS5 and PS15. In addition, bottle S was only fed with syngas. All bottles were placed in symmetrical positions into air bath shakers (150 rpm) at 37 °C. All the bottles were subjected to semi-continuous anaerobic digestion and settled for 1 h before removing 40 mL supernatant and feeding 40 mL fresh substrate everyday. All bottles were operated 45 days and the hydraulic retention time was controlled at 3 days. After feeding the bottles everyday, G5, G15, P5 and P15 were sparged with N_2_ while GS5, GS15, PS5, PS15 and S were sparged with syngas (40% H_2_/40% CO/20% CO_2_), and then closed with rubber stoppers. The pH of all bottles was then adjusted to 9 by adding 4 M sodium hydroxide and the final pH were measured everyday which were all around 8.5 at steady state (Additional file 1: Fig. S1). In all the bottles, 2-bromoethanesulfonate (BES) was added initially to inhibit methanogens. Time curves of organic degradation and syngas consumption were monitored every 3 h in 24 h with one feeding cycle in all bottles.

### Effect of NH_4_^+^-N concentration on conversion efficiency of syngas

The fermentation of BSA produced NH_4_^+^-N, and therefore the effect of NH_4_^+^-N concentration on the conversion efficiency of syngas was investigated by batch experiments. Each bottle (320 mL serum bottles) was inoculated with source culture from the continuous S, and the final working volume was 120 mL with the concentration of anaerobic sludge 5 gVS/L. The NH_4_^+^-N concentrations were set as 0 and 1000 mg/L, respectively, and all the bottles were purged with syngas (40% H_2_/40% CO/20% CO_2_) for 5 min and then closed with rubber stoppers. The above bottles were then incubated in a reciprocating air bath shaker at 37 °C and set at 150 rpm. All the tests were performed in triplicates.

### Microbial community compositions as revealed by high-throughput 16S rRNA gene sequencing

Samples were obtained from all the bottles on day 40 when all the bottles reached steady-state. The names of the nine samples were the same as the names of bottles. The inoculum was also used for microbial analysis. Total genomic DNA of all the samples were extracted from each sample using QIAamp DNA Stool Mini Kit (QIAGEN, 51504) and then used for PCR amplification with primers 515F (5ʹ-GTGCCAGCMGCCGCGGTAA-3ʹ) and 806R (5ʹ-GGACTACVSGGGTATCTAAT-3ʹ). The PCR products were purified, quantified, and used for barcoded libraries preparation and sequenced on an Illumina Miseq platform by Majorbio Bio-Pharm Technology Co. Ltd. (Shanghai, China). The detailed information of DNA extraction, PCR and bioinformatic analysis was described in Additional file 1. All raw sequence data in this study were submitted to NCBI Sequence Read Archive with accession number SUB4897034.

### qPCR analysis

Genomic DNA of the samples obtained in microbial community analysis was also used for qPCR analysis and the total bacteria and acetogens were quantified by qPCR. The primers used for qPCR were as follows: Eub338 (ACT CCT ACG GGA GGC AGC AG)/Eub518(ATT ACC GCG GCT GCT GG) for bacteria and FTHFS-F(GTW TGG GCW AAR GGY GGM GAA GG)/FTHFS-R(GTA TTG DGT YTT RGC CAT ACA) for acetogens [52]. The detailed information is also described in Additional file 1.

### Label-free quantitative proteomic analysis

Four samples (G5, GS5, P5 and PS5) were obtained during the steady states. Total proteins in samples were extracted and separated by polyacrylamide gel electrophoresis (PAGE). And the total protein concentration was determined through the Bradford method [53]. 0.5 μg/μL of peptide of each sample was used for protein identification by liquid chromatograph-mass spectrometer/mass spectrometer (LC-MS/MS) (Thermo Scientific EASY-Nlc II nano liquid chromatograph; Thermo Scientific Q-Exactive mass spectrometer) analysis and label-free differential expression analysis. Raw data were processed using PEAKSStudio version 8.5 (Thermo Scientific ProMass deconvolution software) against NCBI database containing 719029 sequences. Detail information about the bioinformatic analysis can be found in SI.

### Analytical methods

The concentrations of lactate were determined by high-performance liquid chromatography (HPLC) and an Aminex HPX-87H 300 mm × 7.8 mm cation-exchange column (at 60 °C) was used to separate lactate from other compounds (Bio-Rad Laboratories Inc., Hercules, CA, USA) with 5 mM sulphuric acid as the mobile phase (0.6 mL/min). The gas composition was analyzed by a gas chromatography (GC) equipped with a TCD detector. For CO, the carrier gas was He. For H_2_, the carrier gas was changed to N_2_. For CO and H_2_, the temperatures of the injector, detector and oven were 120 °C, 110 °C and 120 °C, respectively. The determination of VFA, soluble protein, soluble carbohydrate, NH_4_^+^-N, and pH were described by our previous study [54]. The analysis of variance (ANOVA) was used to test the significance of results, and *P* < 0.05 was considered to be statistically significant.

## Supplementary information

**Additional file 1.** Figures S1–S8 and Table S1–S6.

## Data Availability

All data generated or analyzed during this study are included in this published article and its additional files.

## Abbreviations

VFA: Volatile fatty acid
AD: Anaerobic digestion
WAS: Waste activated sludge
BSA: Bovine serum albumin
BES: 2-Bromoethanesulfonate
PAGE: Polyacrylamide gel electrophoresis
LC–MS/MS: Liquid chromatograph–mass spectrometer/mass spectrometer
ANOVA: An analysis of variance
