# Supplementary material for: Microbial insights of enhanced anaerobic conversion of syngas into volatile fatty acids by co-fermentation with carbohydrate-rich synthetic wastewater
Source: Biotechnol Biofuels. 2020 Mar 16;13:53. doi: 10.1186/s13068-020-01694-z (PMC7076986; doi:10.1186/s13068-020-01694-z)
Supplement: Supplementary file 1 — Additional file 1. Figures S1–S8 and Table S1–S6. [file 13068_2020_1694_MOESM1_ESM.docx]

Additional information

**Microbial insights of enhanced anaerobic conversion of syngas into volatile fatty acids by co-fermentation with carbohydrate-rich synthetic wastewater**

Chao Liu^1, 2^, Wen Wang^1*^, Sompong O-Thong^2,3^, Ziyi Yang^1^, Shicheng Zhang^2,4^, Guangqing Liu^1^, Gang Luo^2,4*^

1. Biomass Energy and Environmental Engineering Research Center, Beijing University of Chemical Technology, Beijing 100029, China;

2. Shanghai Key Laboratory of Atmospheric Particle Pollution and Prevention (LAP^3^), Department of Environment Science and Engineering, Fudan University, 200433 Shanghai, China;

3. Department of Biology, Faculty of Science, Thaksin University, Phathalung, 93110, Thailand;

4. Shanghai Institute of Pollution Control and Ecological Security, Shanghai 200092, China;

***To whom correspondence should be addressed:***

* Wen Wang’s e-mail: [wangwen@mail.buct.edu.cn](mailto:wangwen@mail.buct.edu.cn), anne_wangwen@163.com (W. Wang);

Tel: +86-010-64429591

Address: Biomass Energy and Environmental Engineering Research Center, Beijing University of Chemical Technology, 100029, Beijing, China

* Gang Luo’s e-mail: gangl@fudan.edu.cn (G. Luo);

Tel: +86-021-65642297

Address: Department of Environmental Science and Engineering, Fudan University, 200438, Shanghai, China

**Effect of acetate and propionate concentration on conversion efficiency of syngas**

The effect of acetate and propionate concentration on the conversion efficiency of syngas was investigated by batch experiments. Each bottle (320 mL serum bottles) was inoculated from a mesophilic anaerobic reactor treating cassava stillage in an ethanol plant, and the final working volume was 120 mL with the concentration of anaerobic sludge 5 gVS/L. The concentrations of acetate and propionate were determined by the products concentration produced from glucose (45 mM acetate and 6 mM propionate in G5 and 48 mM acetate and 67 mM propionate in G15), and all the bottles were purged with syngas (40%H_2_/40%CO/20%CO_2_) for 5 min and then closed with rubber stoppers. The above bottles were then incubated in a reciprocating air bath shaker at 37 ^°^C with 150 rpm. All the tests were performed in triplicates.

**High-throughput sequencing of 16S rRNA genes**

The PCR conditions were as follows: 95 °C for 3 min; 30 cycles of three steps: 95 °C for 30 s, 55 °C for 45 s, and 72 °C for 90 s; a final extension step at 72 °C for 10 min. The PCR products were purified, quantified, and used for barcoded libraries preparation and sequenced on an Illumina Miseq platform. The sequences without exact matches to the forward and reverse primers, with length shorter than 100 bp, and containing any ambiguous base calls were removed by MOTHUR(1). Chimeras were also removed from the sequences by using the Find Chimeras web tool. For all the samples, the numbers of high-quality sequences were between 30561 and 44557 and the average length of the high-quality sequences were between 261 and 280. All the samples were subsampled to 3000 to facilitate the comparison among the samples. The sequences were clustered into operational taxonomic units (OTU) by setting a 0.03 distance limit by MOTHUR program.

**Real-time PCR quantification analysis**

2 μL DNA template, 12.5 μL SybrGreen qPCR master mix, 0.5 μL primers and 9.5 μL water were added to the 20-μL reaction capillary tube. The PCR conditions were as follows: initial denaturation for 10 min at 95 °C, followed by 40 cycles of 15 s at 95 °C and then simultaneous annealing and extension for 60 s at 60 °C. All assays were analyzed in triplicates. Baseline and threshold calculations were undertaken using Realplex software. Standard curves were constructed using a purified DNA template that was amplified by conventional PCR using the corresponding primers. Negative controls using water and positive controls using the corresponding cells were included for each assay.

**Quantitative proteomic analysis**

First, 5g of ice-cold samples was homogenized using Tissue Lyser for three times and for 40s each time. The homogenates were centrifuged at 12000g and 4 °C for 20 min and the supernatant was collected and added with an equal volume of Tris-phenol with 4 min of shaking. The liquid samples were centrifuged at 12000g and 4 °C for 20 min and an equal volume of Benproperine Phosphate was added with 4 min of shaking. Then the liquid samples were centrifuged at 12000g and 4 °C for 20 min, the protein mixtures was precipitated by adding 5× volume of ice-cold ammonium acetate in methanol at -20°C overnight. After centrifugation at 4 °C and 12000g for 20 min, the precipitate was added with 90% acetone in ice and this step was repeated two times, and then the precipitate was suspended in the lysis buffer. After centrifugation at 4 °C and 12000g for 20 min, the proteins in the supernatant were kept and the total protein concentration was determined through the Bradford method(2).

Total protein (150 μg) was taken out of each sample and then the volume was added to 150 μg by adding lysis buffer. The Bond-Breaker^TM^ TCEP solution (TCEP) was added and the concentration was 10mM for 1h at 37 °C. Then Iodoacetamide was added and the concentration was 40mM at dark condition for alkylation at room temperature for 40 min. The liquid sample was diluted seven times by adding 100 mM Triethylammonium bicarbonate buffer (TEAB). Then, each sample was subjected to trypsin digestion with the mass ratio of 50:1 at 37 °C overnight. Final peptide mixture was concentrated in a speed vacuum and supended in 2% CAN, 0,1% TFA. Finally, 0,5μg/μL of peptide of each sample was used for protein identification by liquid chromatograph-mass spectrometer/mass spectrometer (LC-MS/MS) (Thermo Scientific EASY-Nlc II nano liquid chromatograph; Thermo Scientific Q-Exactive mass spectrometer) analysis and lable-free differential expression analysis. For that the sample was loaded onto a C18 reversed-phase column (75μm ×25 cm, thermo, UAS). The mobile phases were composed of A mobile phase (2% ACN, 0.1% FA) and B mobile phase (80% ACN, 0.1% FA) over 120 min at a flow rate of 300 nL/min. Analysis was carried out in a data-dependent acquisition mode, and survey MS1 scans were acquired from 350-1300 m/z. Both full and fragmentation scans were measured in the Q-Exactive^TM^ mass analyser using 70000 and 17500 resolution, respectively. Peptides were fragmented using higher-energy collisional dissociation (HCD). The 20 highest peaks for each full scan were subjected to MS/MS. A dynamic exclusion time of 18s was used to fragment the low abundance compounds. Raw data were processed using PEAKSStudio version 8.5 (Thermo Scientific ProMass deconvolution software) against NCBI database containing 719029 sequences. The search parameters were set as follows: the search of precursor mass tolerance of for identification, a mass tolerance of 10 ppm was used for mass recalibration. The search also included the dynamic modification of methionine oxidation and N-terminal acetylation. For protein identification, precursor mass and fragment mass had initial mass tolerances of 10 ppm and 0.05Da, respectively. For the identification of peptides and proteins, fale discovery rate (FDR) was set to 0.01, and only significant peptides were accepted for the dentification of the protein sample. Each confident protein identification involve at least one unique peptide. For protein quantitation, it was required that a protein contains at least two unique spectra. Differentially expressed proteins were defined based on previous studies(3, 4), and the general criterion for differentially expressed proteins was the fold changes of the proteins ≥1.2 with a P value **<** 0.05. The quantitative protein ratios were weighted and normalized by the median ratio. Differentially expressed proteins were defined based on previous studies (Chen et al. 2016b, Yang et al. 2017). Functional annotations of the proteins were conducted using Blast2GO program against the non-redundant protein database (NR; NCBI). The KEGG database (http://www.genome.jp/kegg/) and COG database (http://www.ncbi.nlm.nih. gov/COG/) were used to annotate and group these identified proteins. The enriched pathways analysis was conducted by the online OmicsBean bioinformatics resource (http://www.omicsbean.com).

**Proteomic profiles changes in syngas and protein-rich co-fermentation system**

The changes of protein expression levels induced by co-fermentation of syngas with BSA in PS5 compared to P5 were characterized by label-free quantitative proteomic technology. 2693 proteins were identified from all the samples. 715 of the 2693 proteins were identified as differentially expressed proteins in PS5 and P5 (Fig.S10), and 400 proteins were up-regulated and 315 proteins were down-regulated. As shown in Table S6, the biological regulation, metabolic process,, and cellular process involved in biological process by GO enrichenment analysis were significantly enriched (P<0.05). In addition, cell part and extracellular involved in cellular component by GO enrichenment analysis were also significantly enriched (P<0.05). The differentially expressed protein by KEGG database were found to be enriched in carbon fixation pathways in prokaryotes (12.9%), pyruvate metabolism (10.8%), and alanine, aspartate and glutamate metabolism (2.9%). The differentially expressed proteins, relating with key enzymes (e.g. glycine reductase, and alanine dehydrogenase) were up-regulated in stickland reaction which can be conduct by glycine and alanine, and acetate was the final product, which was consistent with higher acetate production with syngas addition in PS5.

Fig.S1. Time curves of pH in all continuous bottles during 45 days.


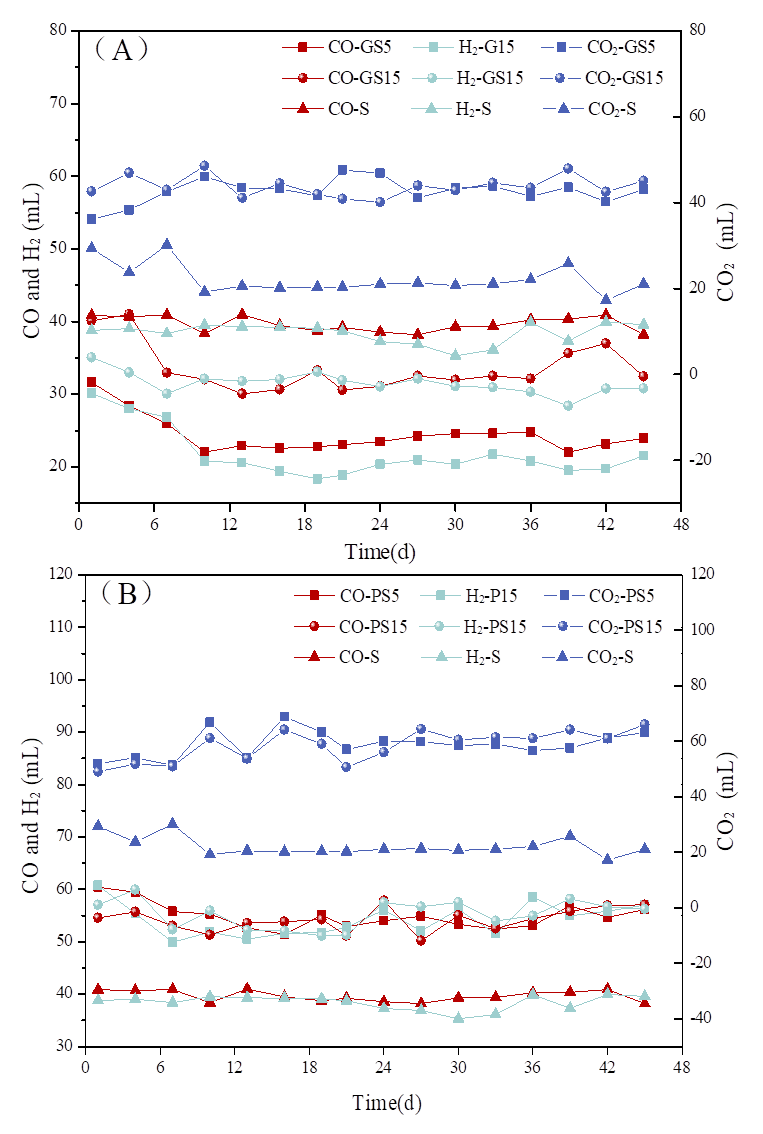


Fig.S2. Time curves of collected gases in continuous bottles during 45 days. The results from bottles with glucose as substrate are shown in (A) B), and the results from bottle with BSA as substrate are shown in (B).

Fig.S3. Consumption of syngas at different concentration of NH_4_^+^-N (0 and 1000 mg/L)


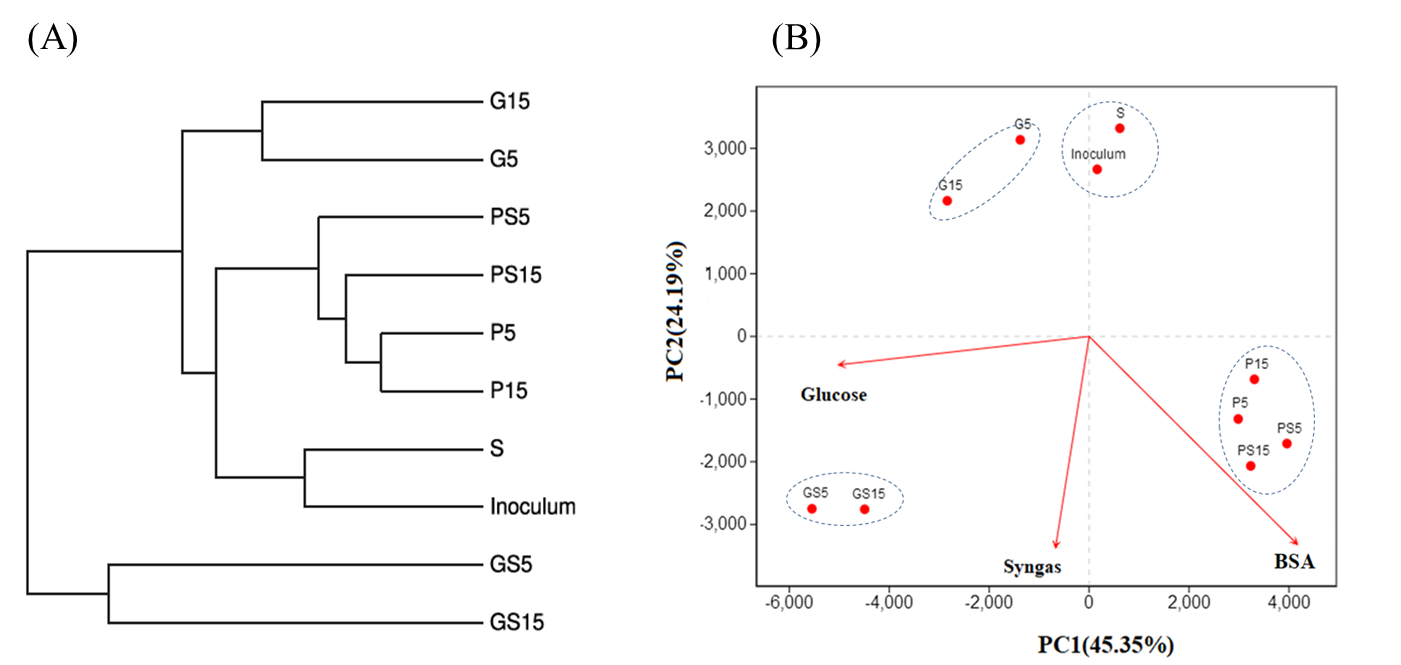


Fig.S4. (A) Rectanglar cladogram based on OTU analysis with Bray-Curtis similarity matrix at 0.03 distance;

(B) PCA analysis of all the samples by 0.03 cutoff.


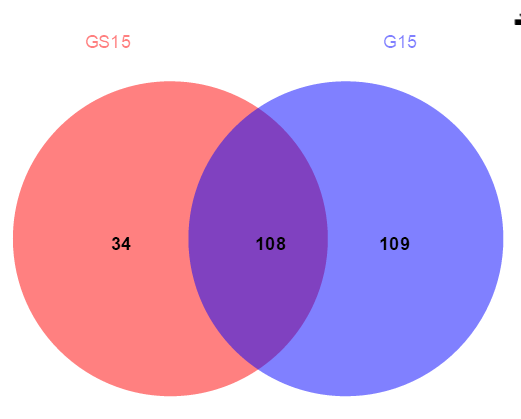

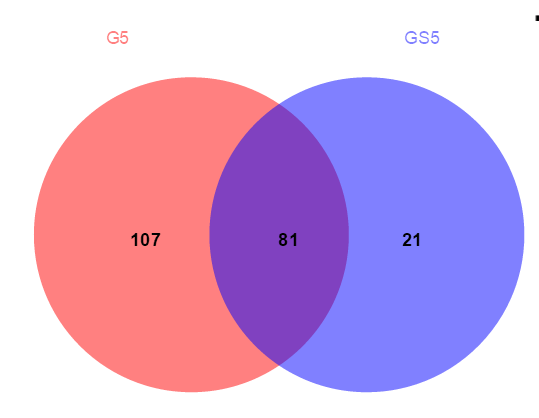

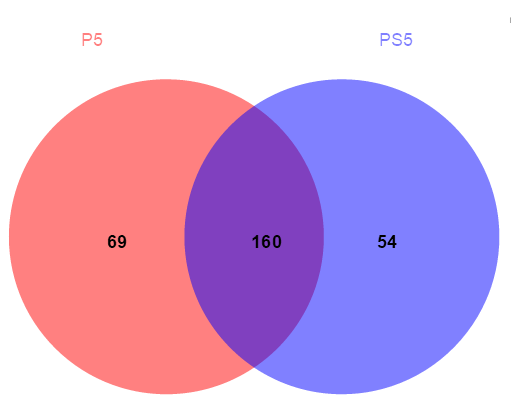

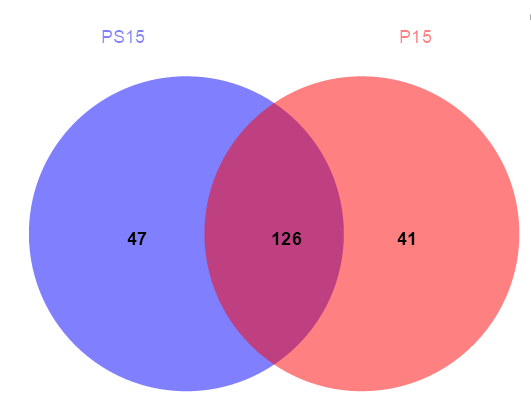


Fig.S5. Venn diagram based on OTU analysis at 0.03 distance.


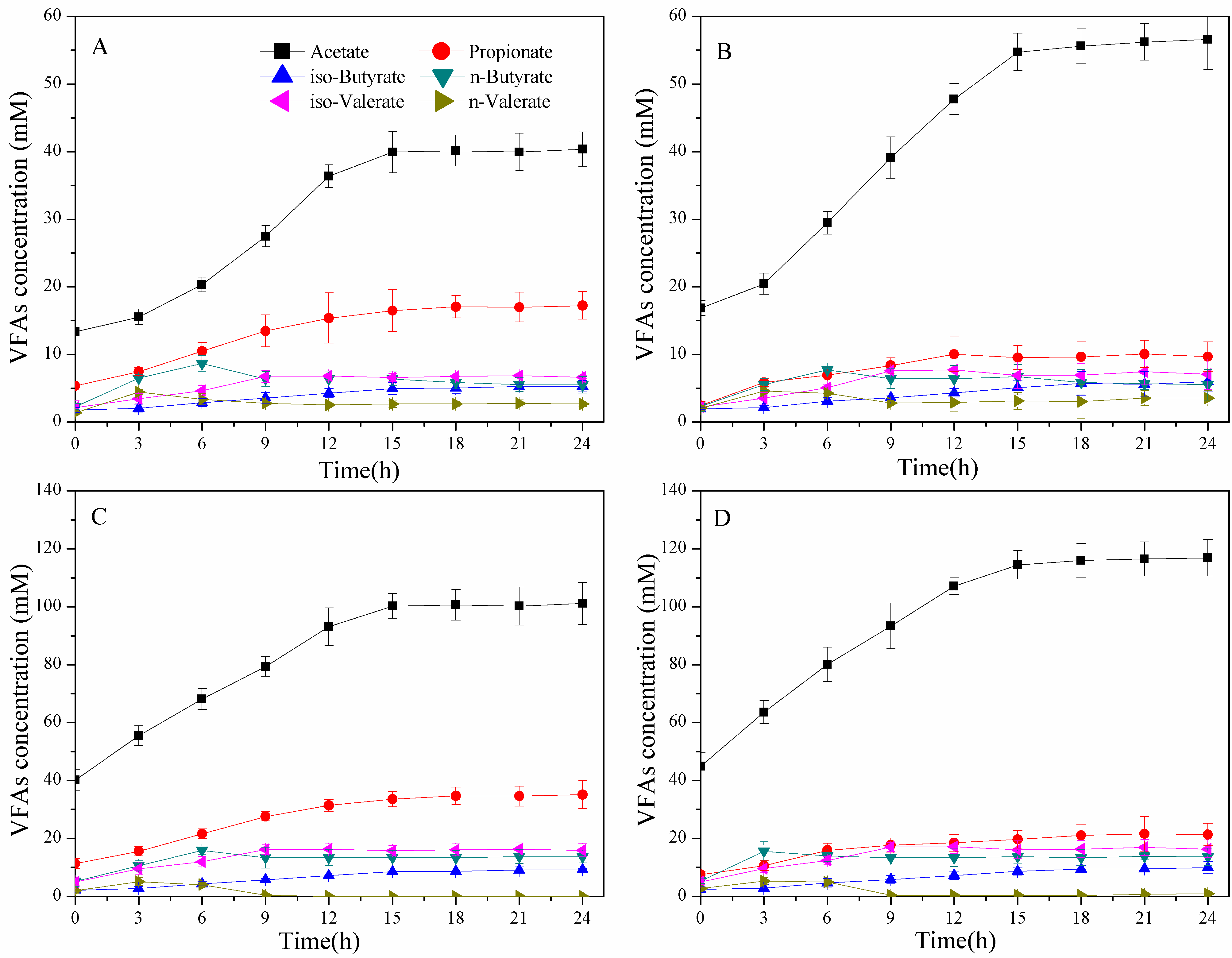


Fig.S6. Time curves of VFAs production during 24h for P5 (A), PS5 (B), P15 (C) and PS15 (D).


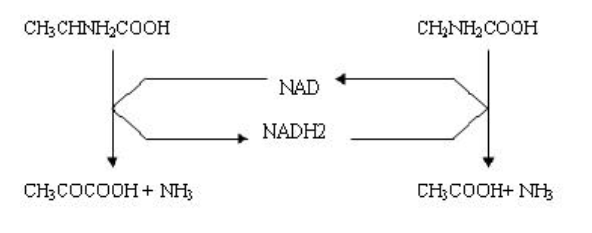


Fig.S7. Stickland degradation pathway of amino acid

Fig.S8. Numbers of up-regulated or down-regulated proteins

Table S1 The significant analysis of CO/H_2_ conversion efficiency among all the reactors

| Reactor | | P-value^a,b^ | |
| --- | --- | --- | --- |
|  |  | CO conversion efficiency (%) | H_2_ conversion efficiency (%) |
| GS5 | GS15 | 6.13E-08 | 6.120E-11 |
|  | PS5 | 1.62E-12 | 1.617E-12 |
|  | PS15 | 1.62E-12 | 1.616E-12 |
|  | S | 3.01E-08 | 2.988E-12 |
| GS15 | PS5 | 1.62E-12 | 2.130E-12 |
|  | PS15 | 1.62E-12 | 1.627E-12 |
|  | S | 4.77E-11 | 1.856E-07 |
| PS5 | PS15 | 5.69E-07 | 5.808E-08 |
|  | S | 1.62E-12 | 2.024E-11 |
| PS15 | S | 1.66E-12 | 1.949E-12 |

^a^ determined by Duncan with One-way ANOVA analysis

^b^ P<0.005: significant

Table S2 The classification and relative abundances of the OTUs which were enriched in co-fermentation bottles

|  | Relative abundance (%) | | Closest match | Identity(%) |
| --- | --- | --- | --- | --- |
|  | I | S |  |  |
| \| OTU1 \| \| --- \| | 9.217 | 11.847 | *Mesotoga infera*(NR_117646.2) | 99% |
| \| OTU2 \| \| --- \| | 0.417 | 0.833 | *uncultured bacterium*(MG803516) | 97% |
| OTU3 | 0.000 | 3.790 | *Clostridium formicoaceticum* (NR_117711.1) | 97% |
| OTU4 | 0.000 | 0.810 | *Natronincola histidinovorans*(NR_026455.1) | 90% |
| OTU5 | 0.000 | 0.643 | *uncultured Clostridia bacterium* (KX672732.1) | 97% |

|  | Relative abundance (%) | | Closest match | Identity(%) |
| --- | --- | --- | --- | --- |
|  | G5 | GS5 |  |  |
| \| OTU1 \| \| --- \| | 15.580 | 21.880 | *Bacterium NLAE-zl-C76* (JQ607775.1) | 99% |
| \| OTU2 \| \| --- \| | 0.8567 | 2.790 | *Bacteroidetes bacterium VNs52* (FJ168485.1) | 95% |
| OTU3 | 1.950 | 2.690 | *uncultured bacterium* (MH147084) | 99% |
| OTU4 | 0.000 | 9.7633 | *Anaerostipes rhamnosivorans* (JX273468.1) | 91% |
| OTU5 | 0.017 | 7.190 | *Halolactibacillus alkaliphilus*(NR_044282.1) | 99% |
| OTU6 | 1.097 | 1.627 | *Lascolabacillus massiliensis*(NR_144720.1) | 97% |
| OTU7 | 0.000 | 0.460 | *Natronincola histidinovorans*(NR_026455.1) | 90% |
| OTU8 | 0.000 | 0.343 | *Clostridium formicoaceticum* (NR_117711.1) | 97% |

|  | Relative abundance (%) | | Closest match | Identity(%) |
| --- | --- | --- | --- | --- |
|  | G15 | GS15 |  |  |
| OTU1 | 0.460 | 2.113 | *Aminobacterium colombiense* (NR_074624.1) | 91% |
| OTU2 | 0.917 | 4.540 | *Mesotoga infera* (NR_117646.2) | 99% |
| OTU3 | 0.387 | 24.353 | *Geofilum rhodophaeum* (KY659246.1) | 97% |
| OTU4 | 0.180 | 1.193 | *Clostridium formicoaceticum* (NR_117711.1) | 97% |

|  | Relative abundance (%) | | Closest match | Identity(%) |
| --- | --- | --- | --- | --- |
|  | P5 | PS5 |  |  |
| OTU1 | 6.083 | 9.753 | *Aminobacterium colombiense* (NR_074624.1) | 99% |
| OTU2 | 14.870 | 26.263 | *Tepidimicrobium ferriphilum* (JQ897432.1) | 91% |
| OTU3 | 5.447 | 9.543 | *Mesotoga infera*(NR_117646.2) | 99% |
| OTU4 | 1.840 | 2.243 | *uncultured bacterium*(MG803516.1) | 97% |
| OTU5 | 0.210 | 0.960 | *Natronincola histidinovorans*(NR_026455.1) | 90% |

|  | Relative abundance (%) | | Closest match | Identity(%) |
| --- | --- | --- | --- | --- |
|  | P15 | PS15 |  |  |
| OTU1 | 15.423 | 18.967 | *Tepidimicrobium ferriphilum* (JQ897432.1) | 91% |
| OTU2 | 0.480 | 1.687 | *Lascolabacillus massiliensis*(NR_144720.1) | 97% |
| OTU3 | 1.623 | 3.457 | *Clostridium sp. MT1*(LK021112.2) | 95% |
| OTU4 | 1.000 | 1.767 | *Natronincola histidinovorans*(NR_026455.1) | 90% |

Table S3 Reported anaerobic syngas-converting bacteria

| Phylum | Species | Reference |
| --- | --- | --- |
| ***Firmicutes*** | *Acetobacterium woodii* | (5, 6) |
|  | *Acetobacterium carbinolicum* | (7) |
|  | *Acetogenium kivui* | (8) |
|  | *Alkalibaculum bacchi* | (9) |
|  | *Bacillus simithii* | (10) |
|  | *Blautia coccoides* | (11) |
|  | *Butyribacterium methylotrophicum* | (12-14) |
|  | *Carboxydothermus hydrogenoformans* | (15, 16) |
|  | *Clostridium aceticum* | (17) |
|  | *Clostridium autoethanogenum* | (18) |
|  | *Clostridium carboxidivorans* | (19, 20) |
|  | *Clostridium ljungdahlii* | (21) |
|  | *Clostridium thermoaceticum* | (22) |
|  | *Clostridium formicoaceticum* | (23) |
|  | *Clostridium magnum* | (24) |
|  | *Clostridium mayombei* | (25) |
|  | *Clostridium thermoautotrophicum* | (23) |
|  | *Desulfotomaculum orientis* | (26) |
|  | *Desulfotomaculum kutznetsovii* | (27) |
|  | *Desulfotomaculum thermobenzoicum* | (27) |
|  | *Desulfotomaculum carboxydivorans* | (28) |
|  | *Eubacterium limosum* | (29) |
|  | *Moorella thermoacetica* | (22, 30) |
|  | *Moorella glycerini* | (31) |
|  | *Moorella mulderi* | (32) |
|  | *Moorella thermoautotrophica* | (33) |
|  | *Marvinbryantia formatexigens* | (34) |
|  | *Natroniella acetigena* | (35) |
|  | *Natronoincola histidinovorans* | (36) |
|  | *Peotostreotococcus productus* | (37) |
| [***Proteobacteria***](https://en.wikipedia.org/wiki/Proteobacteria) | *Rhodobacter sphaeroides* | (38-40) |
|  | *Rhodocyclus gelatinosus* | (41) |
|  | *Rhodopseudomonas capsulate* | (42) |
|  | *Rhodopseudomonas gelatinosa* | (43-45) |
|  | *Rhodopseudomonas palustris* | (46-48) |
|  | *Rhodospirillum rubrum* | (49-53) |
|  | *Rubrivivax gelatinosus* | (54, 55) |
|  | *Sulfurspirillum carboxydovorans* | (56) |
|  | *Desulfovibrio vulgaris* | (57) |
|  | *Citrobacter amalonaticus* | (58, 59) |

Table S4 Representative sequences of the OTUs enriched in co-fermentation

|  | OTU name | Representative sequences of the selected OTUs |
| --- | --- | --- |
| ***GS5 vs G5*** | OTU1 | TACGTAGGGGGCGAGCGTTACCCGGAATCACTGGGCGTAAAGGGAGCGTAGGTGGCCTAACATGTCAACTGTGAAAACCCGGAGCTCAACTCCGGACTTGCAGTTGAAACTGGTAGGCTTGAGGACGGTAGAGGAAGACGGAACTGCCAGTGTAGGGGTAAAATCCTTAGATATTGGCAGGAACGCCGGTGACGAAGGTGGTTTTCTGGGCCGGTTCTGACACTGATGCTCGAAAGCCAGGGGAGCGAACGGGATTAGATACCCGTGTAGTCC |
|  | OTU2 | TACGGAGGATGCGAGCGTTATCCGGATTTATTGGGTTTAAAGGGTGCGTAGGCTGGTAATTAAGTCAGTGGTGAAATACTCCGGCTCAACCGGGGGGCTGCCGTTGATACTGGTTACCTTGAGTTTAGATGATGTGGGCGGAATGTGTCATGTAGCGGTGAAATGCATAGAGATGACACAGAACACCGATAGCGAAGGCAGCTCACAAATCTATTACTGACGCTGATGCACGAAAGCGTGGGGATCAAACAGGATTAGATACCCGGGTAGTCC |
|  | OTU3 | TACGGAGGGTGCGAGCGTTATTCGGAATTACTGGGCGTAAAGCGCGTGTAGGCGGGGGGACAAGTCTGATGTGAAAGCCCTGGGCTTAACCTGGGAAGCGCATTGGAAACTGTTTTTCTTGAGTACTGGAGAGGAAGGGGGAATTCCCGGTGTAGAGGTGAAATTCGTAGAGATCGGGAGGAATACCAGTGGCGTAGGCGCCCTTCTGGACGGTAACTGACGCTGAGACGCGAAAGCGTGGGGAGCAAACAGGATTAGATACCCGTGTAGTCC |
|  | OTU4 | TGTGAAAAGCCCGGTGGCAGTATAGGATGGTCCCGCGTCTGATTAGCTGGTTGGCGGGGTACATCAGTAGCCGGCTTGAGAGAGTGATGACGGCCACATTGGGACTGAGACACGGCCCAAACTCCTACGGGAGGCAGCAGTGGGGAATATGGTGCACAATGGGGGAAACCCTGATGCAGCGACGCCGCGTGAGTGAAGAAGTATTTCGGTATGTAAAGCTCTATCAGCAGGGAAGAAATAAGACGGTACCTGACTAAGA |
|  | OTU5 | TACGTAGGGGGCAAGCGTTGTCCGGAATTATTGGGCGTAAAGCGCGCGCAGGCGGTCCTTTAAGTCTGATGTGAAATCTTGCGGCTCAACCGCAAGCGGTCATTGGAAACTGGGGGACTTGAGTACAGAAGAGGAGAGTGGAATTCCATGTGTAGCGGTGAAATGCGTAGATATATGGAGGAACACCAGTGGCGAAGGCGACTCTCTGGTCTGTAACTGACGCTGAGGCGCGAAAGCGTGGGGAGCAAACAGGATTAGATACCCGGGTAGTCC |
|  | OTU6 | TACGGAGGATCCGAGCGTTATCCGGATTTATTGGGTTTAAAGGGTGCTCAGGCGGGGGATTAAGTCGGCGGTGAAATTTTGCAGCTCAACTGTAAAAGTGCCATCGATACTGGTTTTCTTGAGTGTGGATGAAGTAGGCGGAATTTGTGGTGTAGCGGTGAAATGCATAGATATCACGAGGAACTCCGATTGCGCAGGCAGCTTACTAGGCCATAACTGACGCTCAGGCACGAAAGCGTGGGGATCAAACAGGATTAGATACCCGTGTAGTCC |
|  | OTU7 | TACGTAGGGGGCAAGCGTTATCCGGAATTATTGGGCGTAAAGGGTGCGTAGGCGGCCAATAAAGTAAGAGGTGAAATCCCAGGGCTCAACCCTGGTCAGCCTTTGAAACTTAATGGCTTGAGTGCAGGAGAGGAGAGTGGAATTCCTAGTGTAGCGGTGAAATGCGTAGATATTAGGAGGAACACCAGTGGCGAAGGCGACTCTCTGGACTGTAACTGACGCTGAGGCACGAAAGTGTGGGTAGCAAACAGGATTAGATACCCGGGTAGTCC |
|  | OUT8 | TACGTAGGGGGCAAGCGTTATCCGGAATCACTGGGCGTAAAGGGTGCGTAGGCGGCCAAACAAGTCAGGGGTGAAGGGCTACGGCTCAACCGTAGTAAGCCTTGGAAACTTATTGGCTTGAGTGCAGGAGAGGAGAGTGGAATTCCTAGTGTAGCGGTGAAATGCGTAGATATTAGGAGGAACACCAGTGGCGAAGGCGACTCTCTGGACTGTAACTGACGCTGAGGCACGAAAGCGTGGGGAGCGAACAGGATTAGATACCCGTGTAGTCC |
| ***GS15 vs G15*** | OTU1 | TACGTAGGGTGCGAGCGTTGTCCGGAATTACTGGGCGGAAAGGGCGCGCCGGCTGTGCTGCAAGTCAGCTGTTAAATGTCTGGGCTTAACCCGGGCATGCGGTTGAGACTGCGGTGCTGGAGTGCTGTAGAGGCAAGTGGAATTCCCAGTGTAGCGGTGAAATGCGTAGATATTGGGAAGAACACCGGTGGCGAAGGCGACTTGCTGGGCAGCAACTGACGCTGAGGCGCGAAAGCATGGGTAGCGAACAGGATTAGATACCCGTGTAGTCC |
|  | OTU2 | TACGTAGGGGGCGAGCGTTACCCGGAATCACTGGGCGTAAAGGGAGCGTAGGTGGCCTAACATGTCAACTGTGAAAACCCGGAGCTCAACTCCGGACTTGCAGTTGAAACTGGTAGGCTTGAGGACGGTAGAGGAAGACGGAACTGCCAGTGTAGGGGTAAAATCCTTAGATATTGGCAGGAACGCCGGTGACGAAGGTGGTTTTCTGGGCCGGTTCTGACACTGATGCTCGAAAGCCAGGGGAGCGAACGGGATTAGATACCCGTGTAGTCC |
|  | OTU3 | TACGGAGGATCCGAGCGTTATCCGGATTTATTGGGTTTAAAGGGTGCGTAGGCGGACTAGTAAGTCAGTGGTGAAATCCTGCAGCTTAACTGTAGAATTGCCGTTGATACTGTTAGTCTTGAGTACATTTGAGGTAGGCGGAATGTGGTGTGTAGCGGTGAAATGCATAGATATACCACAGAACACCGATTGCGCAGGCAGCTTACTAAACCATAACTGACGCTCAAGCACGAAGGCGTGGGGATCAAACAGGATTAGATACCCGGGTAGTCC |
|  | OTU4 | TACGTAGGGGGCAAGCGTTATCCGGAATCACTGGGCGTAAAGGGTGCGTAGGCGGCCAAACAAGTCAGGGGTGAAGGGCTACGGCTCAACCGTAGTAAGCCTTGGAAACTTATTGGCTTGAGTGCAGGAGAGGAGAGTGGAATTCCTAGTGTAGCGGTGAAATGCGTAGATATTAGGAGGAACACCAGTGGCGAAGGCGACTCTCTGGACTGTAACTGACGCTGAGGCACGAAAGCGTGGGGAGCGAACAGGATTAGATACCCGTGTAGTCC |
| ***PS5 vs P5*** | OTU1 | TACGTAGGGTGCGAGCGTTGTCCGGAATTACTGGGCGGAAAGGGCGCGCCGGCTGTGCTGCAAGTCAGCTGTTAAATGTCTGGGCTTAACCCGGGCATGCGGTTGAGACTGCGGTGCTGGAGTGCTGTAGAGGCAAGTGGAATTCCCAGTGTAGCGGTGAAATGCGTAGATATTGGGAAGAACACCGGTGGCGAAGGCGACTTGCTGGGCAGCAACTGACGCTGAGGCGCGAAAGCATGGGTAGCGAACAGGATTAGATACCCGTGTAGTCC |
|  | OTU2 | TACGTAGGGGGCGAGCGTTGTCCGGAATTACTGGGCGTAAAGGGTACGCAGGCGGCCATGTAAGTCAGATGTGAAAGGTCACGGCTTAACCGTGGCAAGCATTTGAAACTGTGTGGCTTGAGTTAAGGAGAGGAAAGTGGAATTCCTGGTGTAGCGGTGAAATGCGTAGATATCAGGAGGAATATCAGTGGCGAAGGCGACTTTCTGGACTTAAACTGACGCTGAGGTACGAAAGCGTGGGTAGCAAACAGGATTAGATACCCGTGTAGTCC |
|  | OTU3 | TACGTAGGGGGCGAGCGTTACCCGGAATCACTGGGCGTAAAGGGAGCGTAGGTGGCCTAACATGTCAACTGTGAAAACCCGGAGCTCAACTCCGGACTTGCAGTTGAAACTGGTAGGCTTGAGGACGGTAGAGGAAGACGGAACTGCCAGTGTAGGGGTAAAATCCTTAGATATTGGCAGGAACGCCGGTGACGAAGGTGGTTTTCTGGGCCGGTTCTGACACTGATGCTCGAAAGCCAGGGGAGCGAACGGGATTAGATACCCGTGTAGTCC |
|  | OTU4 | TACGAGGGGGGCAAGCATTATGCGGAATCATTGGGCGTACAGGGTGTGTAGGCGGCATGTTAAGTATGTTGTTAAAGACTCCGGCCTAACCGGAGATGGGCAGCGGAAACTGGCATACTAGAGGTTGGAAGAGAGAAGCGGAATTCTCGGTGTAGCGGTAAAATGCGTAGATATCGAGAGGAACACCGATGGCGAAGGCAGCTTCTTGGTCCATACCTGACGCTGAAACACGAAAGCGTGGGGAGCAAACGGGATTAGATACCCGTGTAGTCC |
|  | OTU5 | TACGTAGGGGGCAAGCGTTATCCGGAATTATTGGGCGTAAAGGGTGCGTAGGCGGCCAATAAAGTAAGAGGTGAAATCCCAGGGCTCAACCCTGGTCAGCCTTTGAAACTTAATGGCTTGAGTGCAGGAGAGGAGAGTGGAATTCCTAGTGTAGCGGTGAAATGCGTAGATATTAGGAGGAACACCAGTGGCGAAGGCGACTCTCTGGACTGTAACTGACGCTGAGGCACGAAAGTGTGGGTAGCAAACAGGATTAGATACCCGGGTAGTCC |
| ***PS15 vs P15*** | OTU1 | TACGTAGGGGGCGAGCGTTGTCCGGAATTACTGGGCGTAAAGGGTACGCAGGCGGCCATGTAAGTCAGATGTGAAAGGTCACGGCTTAACCGTGGCAAGCATTTGAAACTGTGTGGCTTGAGTTAAGGAGAGGAAAGTGGAATTCCTGGTGTAGCGGTGAAATGCGTAGATATCAGGAGGAATATCAGTGGCGAAGGCGACTTTCTGGACTTAAACTGACGCTGAGGTACGAAAGCGTGGGTAGCAAACAGGATTAGATACCCGTGTAGTCC |
|  | OTU2 | TACGGAGGATCCGAGCGTTATCCGGATTTATTGGGTTTAAAGGGTGCTCAGGCGGGGGATTAAGTCGGCGGTGAAATTTTGCAGCTCAACTGTAAAAGTGCCATCGATACTGGTTTTCTTGAGTGTGGATGAAGTAGGCGGAATTTGTGGTGTAGCGGTGAAATGCATAGATATCACGAGGAACTCCGATTGCGCAGGCAGCTTACTAGGCCATAACTGACGCTCAGGCACGAAAGCGTGGGGATCAAACAGGATTAGATACCCGTGTAGTCC |
|  | OTU3 | TACGTAGGGGGCGAGCGTTGTCCGGAATTATTGGGCGTAAAGGGTTCGCAGGCGGCCAGATAAGTCAGATGTGAAATCTCACGGCTTAACCGTGATAAGCATTTGAAACTGTATGGCTTGAGTTAAGGAGAGGAAAGTGGAATTCCTGGTGTAGCGGTGAAATGCGTAGATATCAGGAGGAATATCAGTGGCGAAGGCGACTTTCTGGACTTAAACTGACGCTGAGGTACGAAAGCGTGGGTAGCAAACAGGATTAGATACCCGTGTAGTCC |
|  | OTU4 | TACGTAGGGGGCAAGCGTTATCCGGAATTATTGGGCGTAAAGGGTGCGTAGGCGGCCAATAAAGTAAGAGGTGAAATCCCAGGGCTCAACCCTGGTCAGCCTTTGAAACTTAATGGCTTGAGTGCAGGAGAGGAGAGTGGAATTCCTAGTGTAGCGGTGAAATGCGTAGATATTAGGAGGAACACCAGTGGCGAAGGCGACTCTCTGGACTGTAACTGACGCTGAGGCACGAAAGTGTGGGTAGCAAACAGGATTAGATACCCGGGTAGTCC |
| ***S vs I*** | OTU1 | TACGTAGGGGGCGAGCGTTACCCGGAATCACTGGGCGTAAAGGGAGCGTAGGTGGCCTAACATGTCAACTGTGAAAACCCGGAGCTCAACTCCGGACTTGCAGTTGAAACTGGTAGGCTTGAGGACGGTAGAGGAAGACGGAACTGCCAGTGTAGGGGTAAAATCCTTAGATATTGGCAGGAACGCCGGTGACGAAGGTGGTTTTCTGGGCCGGTTCTGACACTGATGCTCGAAAGCCAGGGGAGCGAACGGGATTAGATACCCGTGTAGTCC |
|  | OTU2 | TACGAGGGGGGCAAGCATTATGCGGAATCATTGGGCGTACAGGGTGTGTAGGCGGCATGTTAAGTATGTTGTTAAAGACTCCGGCCTAACCGGAGATGGGCAGCGGAAACTGGCATACTAGAGGTTGGAAGAGAGAAGCGGAATTCTCGGTGTAGCGGTAAAATGCGTAGATATCGAGAGGAACACCGATGGCGAAGGCAGCTTCTTGGTCCATACCTGACGCTGAAACACGAAAGCGTGGGGAGCAAACGGGATTAGATACCCGTGTAGTCC |
|  | OTU3 | TACGTAGGGGGCAAGCGTTATCCGGAATCACTGGGCGTAAAGGGTGCGTAGGCGGCCAAACAAGTCAGGGGTGAAGGGCTACGGCTCAACCGTAGTAAGCCTTGGAAACTTATTGGCTTGAGTGCAGGAGAGGAGAGTGGAATTCCTAGTGTAGCGGTGAAATGCGTAGATATTAGGAGGAACACCAGTGGCGAAGGCGACTCTCTGGACTGTAACTGACGCTGAGGCACGAAAGCGTGGGGAGCGAACAGGATTAGATACCCGTGTAGTCC |
|  | OTU4 | TACGTAGGGGGCAAGCGTTATCCGGAATTATTGGGCGTAAAGGGTGCGTAGGCGGCCAATAAAGTAAGAGGTGAAATCCCAGGGCTCAACCCTGGTCAGCCTTTGAAACTTAATGGCTTGAGTGCAGGAGAGGAGAGTGGAATTCCTAGTGTAGCGGTGAAATGCGTAGATATTAGGAGGAACACCAGTGGCGAAGGCGACTCTCTGGACTGTAACTGACGCTGAGGCACGAAAGTGTGGGTAGCAAACAGGATTAGATACCCGGGTAGTCC |
|  | OTU5 | TACGTAGGGGGCAAGCGTTGTCCGGAATTATTGGGCGTAAAGGGTGCGTAGGCGGCCTTTTAATTCAGACGTGAAAGCCCACGGCTTAACCGTGGGATTGCATTTGAAACTGGAAGGCTTGAGTATCGGAGAGGGTAGTGGAATTCCCAGTGTAGCGGTGAAATGCGTAGATATTGGGAGGAACACCAGTGGCGAAGGCGACTACCTGGACGAAAACTGACGCTGAGGCACGAAAGCTAGGGGAGCAAACAGGATTAGATACCCGGGTAGTC |

Table S5 GO enrichment analysis in GS5 compared with G5

| name | namespace | diff_number |
| --- | --- | --- |
| carbon utilization | biological_process | 14\|222 |
| structural molecule activity | molecular_function | 21\|222 |
| organelle | cellular_component | 18\|222 |
| membrane | cellular_component | 17\|222 |
| antioxidant activity | molecular_function | 2\|222 |
| cellular component organization or biogenesis | biological_process | 18\|222 |
| cell part | cellular_component | 94\|222 |
| binding | molecular_function | 133\|222 |
| catalytic activity | molecular_function | 147\|222 |
| response to stimulus | biological_process | 3\|222 |

Table S6 KEGG pathway enrichment analysis in GS5 compared with G5

| Pathway ID | pathway_name | diff_number_of_accs | p_value |
| --- | --- | --- | --- |
| ko00670 | One carbon pool by folate | 6\|166 | 0.023382472 |
| ko05230 | Central carbon metabolism in cancer | 2\|166 | 0.029627402 |
| ko03010 | Ribosome | 11\|166 | 0.000102502 |
| ko00071 | Fatty acid degradation | 12\|166 | 0.000870528 |
| ko00350 | Tyrosine metabolism | 9\|166 | 0.001078456 |
| ko04930 | Type II diabetes mellitus | 2\|166 | 0.029627402 |
| ko01230 | Biosynthesis of amino acids | 39\|166 | 0.00082896 |
| ko00010 | Glycolysis / Gluconeogenesis | 36\|166 | 0.000682954 |
| ko04066 | HIF-1 signaling pathway | 9\|166 | 0.041331999 |
| ko02060 | Phosphotransferase system (PTS) | 2\|166 | 0.029627402 |
| ko00710 | Carbon fixation in photosynthetic organisms | 17\|166 | 0.001276214 |
| ko00520 | Amino sugar and nucleotide sugar metabolism | 3\|166 | 0.0348628 |
| ko00626 | Naphthalene degradation | 8\|166 | 0.000354201 |
| ko00650 | Butanoate metabolism | 18\|166 | 0.034318968 |
| ko01210 | 2-Oxocarboxylic acid metabolism | 11\|166 | 0.03240526 |
| ko00051 | Fructose and mannose metabolism | 7\|166 | 0.036496279 |
| ko00625 | Chloroalkane and chloroalkene degradation | 8\|166 | 0.000354201 |
| ko01200 | Carbon metabolism | 66\|166 | 0.050406084 |
| ko01220 | Degradation of aromatic compounds | 8\|166 | 0.001230124 |
| ko01130 | Biosynthesis of antibiotics | 60\|166 | 0.002057756 |
| ko05010 | Alzheimer disease | 7\|166 | 0.006104199 |

**Reference:**

1. Luo G, Wang W, Angelidaki I. 2013. Anaerobic digestion for simultaneous sewage sludge treatment and CO biomethanation: process performance and microbial ecology. Environmental science & technology 47:10685-10693.

2. Sengupta D, Kannan M, Reddy AR. 2011. A root proteomics-based insight reveals dynamic regulation of root proteins under progressive drought stress and recovery in Vigna radiata (L.) Wilczek. Planta 233:1111-1127.

3. Chen T, Zhang L, Shang H, Liu S, Peng J, Gong W, Shi Y, Zhang S, Li J, Gong J. 2016. iTRAQ-based quantitative proteomic analysis of cotton roots and leaves reveals pathways associated with salt stress. PLoS One 11:e0148487.

4. Yang A, Yu L, Chen Z, Zhang S, Shi J, Zhao X, Yang Y, Hu D, Song B. 2017. Label-free quantitative proteomic analysis of chitosan oligosaccharide-treated rice infected with southern rice black-streaked dwarf virus. Viruses 9:115.

5. Sharak Genthner BR, Bryant MP. 1987. Additional characteristics of one-carbon-compound utilization by Eubacterium limosum and Acetobacterium woodii. Applied & Environmental Microbiology 492-493:471-6.

6. Ragsdale SW, Ljungdahl LG, Dervartanian DV. 1983. Isolation of carbon monoxide dehydrogenase from Acetobacterium woodii and comparison of its properties with those of the Clostridium thermoaceticum enzyme. Journal of Bacteriology 155:1224-37.

7. Eichler B, Schink B. 1984. Oxidation of primary aliphatic alcohols by Acetobacterium carbinolicum sp. nov., a homoacetogenic anaerobe. Archives of Microbiology 140:147-152.

8. Gaddy JL. 2000. Biological production of ethanol from waste gases with Clostridium ljungdahlii. US.

9. Allen TD, Caldwell ME, Lawson PA, Huhnke RL, Tanner RS. 2010. Alkalibaculum bacchi gen. nov., sp. nov., a CO-oxidizing, ethanol-producing acetogen isolated from livestock-impacted soil. International Journal of Systematic & Evolutionary Microbiology 60:2483-2489.

10. Grady JL, Chen GJ. 1998. Bioconversion of waste biomass to useful products.

11. Liu C, Jianzheng LI, Zhang Y, Zhang Y, You L, Liu D. 2016. Sub-culturing of novel Blautia coccoides GA-1 and acetate synthesis from H_2/CO_2 in subcultures. Ciesc Journal.

12. Grethlein AJ, Worden RM, Jain MK, Datta R. 1990. Continuous production of mixed alcohols and acids from carbon monoxide. Applied Biochemistry and Biotechnology 24-25:875-884.

13. Chatterjee S, Grethlein AJ, Worden RM, Jain MK. 1996. Evaluation of support matrices for an immobilized cell gas lift reactor for fermentation of coal derived synthesis gas. Journal of Fermentation & Bioengineering 81:158-162.

14. Heiskanen H, Virkajärvi I, Viikari L. 2007. The effect of syngas composition on the growth and product formation of Butyribacterium methylotrophicum. Enzyme & Microbial Technology 41:362-367.

15. Svetlichny VA, Sokolova TG, Gerhardt M, Ringpfeil M, Kostrikina NA, Zavarzin GA. 1991. Carboxydothermus hydrogenoformans gen. nov., sp. nov., a CO-utilizing Thermophilic Anaerobic Bacterium from Hydrothermal Environments of Kunashir Island. Systematic & Applied Microbiology 14:254–260.

16. Wu M, Ren Q, Durkin AS, Daugherty SC, Brinkac LM, Dodson RJ, Madupu R, Sullivan SA, Kolonay JF, Haft DH. 2006. Correction: Life in Hot Carbon Monoxide: The Complete Genome Sequence of Carboxydothermus hydrogenoformans Z-2901. Plos Genetics 1:: e65.

17. Jiahuey S, Kamaruddin AH, Long WS, Najafpour G. 2007. Clostridium aceticum - a potential organism in catalyzing carbon monoxide to acetic acid: application of response surface methodology. Enzyme & Microbial Technology 40:1234-1243.

18. Abrini J, Naveau H, Nyns EJ. 1994. Clostridium autoethanogenum , sp. nov., an anaerobic bacterium that produces ethanol from carbon monoxide. Archives of Microbiology 161:345-351.

19. Bruant G, Lévesque MJ, Peter C, Guiot SR, Masson L. 2010. Genomic Analysis of Carbon Monoxide Utilization and Butanol Production by Clostridium carboxidivorans Strain P7T. Plos One 5:e13033.

20. Lewis RS, Tanner RS, Huhnke RL. 2007. Indirect or direct fermentation of biomass to fuel alcohol. US.

21. Gaddy. 2000. Biological production of ethanol from waste gases with Clostridium ljungdahlii. Fayettevillear Us 6136577.

22. Kerby R, Zeikus JG. 1983. Growth of Clostridium thermoaceticum on H 2 /CO 2 or CO as energy source. Current Microbiology 8:27-30.

23. Xiao N, Chen Y, Ren H. 2013. Altering protein conformation to improve fermentative hydrogen production from protein wastewater. Water Research 47:5700-5707.

24. Schink B. 1984. Clostridium magnum sp. nov., a non-autotrophic homoacetogenic bacterium. Archives of Microbiology 137:250-255.

25. Kane M, Brauman A, Breznak J. 1991. Clostridium mayombei sp. nov., an H2/CO2 acetogenic bacterium from the gut of the African soil-feeding termite, Cubitermes speciosus. Archives of Microbiology 156:99-104.

26. Zheng X, Su Y, Li X, Xiao N, Wang D, Chen Y. 2013. Pyrosequencing Reveals the Key Microorganisms Involved in Sludge Alkaline Fermentation for Efficient Short-Chain Fatty Acids Production. Environmental Science & Technology 47:4262-4268.

27. Parshina SN, Kijlstra S, Henstra AM, Sipma J, Plugge CM, Stams AJ. 2005. Carbon monoxide conversion by thermophilic sulfate-reducing bacteria in pure culture and in co-culture with Carboxydothermus hydrogenoformans. Applied Microbiology and Biotechnology 68:390-396.

28. Parshina SN, Sipma J, Nakashimada Y, Henstra AM, Smidt H, Lysenko AM, Lens PN, Lettinga G, Stams AJ. 2005. Desulfotomaculum carboxydivorans sp. nov., a novel sulfate-reducing bacterium capable of growth at 100% CO. International Journal of Systematic & Evolutionary Microbiology 55:2159-65.

29. Chang NS, Kim OH, Kim BH, Shin PK, Sung AC, Lovitt RW. 1998. CO fermentation of Eubacterium limosum KIST612. Journal of Microbiology & Biotechnology 8:134-140.

30. Daniel SL, Hsu T, Dean SI, Drake HL. 1990. Characterization of the H2-and CO-dependent chemolithotrophic potentials of the acetogens Clostridium thermoaceticum and Acetogenium kivui. J Bacteriol. Journal of Bacteriology 172:4464-71.

31. Lin P-Y, Whang L-M, Wu Y-R, Ren W-J, Hsiao C-J, Li S-L, Chang J-S. 2007. Biological hydrogen production of the genus Clostridium: Metabolic study and mathematical model simulation. International Journal of Hydrogen Energy 32:1728-1735.

32. Ryan P, Forbes C, Colleran E. 2008. Investigation of the diversity of homoacetogenic bacteria in mesophilic and thermophilic anaerobic sludges using the formyltetrahydrofolate synthetase gene. Water Science And Technology 57:675-680.

33. Leang C, Ueki T, Nevin KP, Lovley DR. 2013. A Genetic System for Clostridium ljungdahlii: a Chassis for Autotrophic Production of Biocommodities and a Model Homoacetogen. Applied and Environmental Microbiology 79:1102-1109.

34. Takai K, Moser DP, Onstott TC, Spoelstra N, Pfiffner SM, Dohnalkova A, Fredrickson JK. 2001. Alkaliphilus transvaalensis gen. nov., sp. nov., an extremely alkaliphilic bacterium isolated from a deep South African gold mine. International Journal of Systematic and Evolutionary Microbiology 51:1245-1256.

35. Liu C, Li J, Zhang Y, Philip A, Shi E, Chi X, Meng J. 2015. Influence of glucose fermentation on CO2 assimilation to acetate in homoacetogen Blautia coccoides GA-1. Journal of Industrial Microbiology & Biotechnology 42:1217-1224.

36. Wang D, Zeng G, Chen Y, Li X. 2015. Effect of polyhydroxyalkanoates on dark fermentative hydrogen production from waste activated sludge. Water research 73:311-322.

37. Lorowitz WH, Bryant MP. 1984. Peptostreptococcus productus strain that grows rapidly with CO as the energy source. Applied & Environmental Microbiology 47:961-4.

38. Adelroth P, Ek MS, Mitchell DM, Gennis RB, Brzezinski P. 1997. Glutamate 286 in cytochrome aa3 from Rhodobacter sphaeroides is involved in proton uptake during the reaction of the fully-reduced enzyme with dioxygen. Biochemistry 36:13824-13829.

39. Mitchell DM, Müller JD, Gennis RB, Nienhaus GU. 1996. FTIR study of conformational substates in the CO adduct of cytochrome c oxidase from Rhodobacter sphaeroides. Biochemistry 35:16782-8.

40. Miksovska J, Gennis RB, Larsen RW. 2006. Thermodynamics of carbon monoxide photodissociation from the fully reduced cytochrome aa3 oxidase from Rb. sphaeroides. Biochimica Et Biophysica Acta 1757:182-188.

41. Maness PC, Weaver PF. 1999. Biological H2 from fuel gases and from H2O. Usdoe Hydrogen Program Review.

42. Jouanneau Y, Kelley BC, Berlier Y, Lespinat PA, Vignais PM. 1980. Continuous monitoring, by mass spectrometry, of H2 production and recycling in Rhodopseudomonas capsulata. Journal of Bacteriology 143:628-36.

43. Dashekvicz MP, Uffen RL. 1979. NOTES: Identification of a Carbon Monoxide-Metabolizing Bacterium as a Strain of Rhodopseudomonas gelatinosa (Molisch) van Niel. International Journal of Systematic Bacteriology 29:145-148.

44. Wakim BT, Uffen RL. 1983. Membrane association of the carbon monoxide oxidation system in Rhodopseudomonas gelatinosa. Journal of Bacteriology 153:571-3.

45. Uffen RL. 1983. Metabolism of carbon monoxide by Rhodopseudomonas gelatinosa: Cell growth and properties of the oxidation system. Journal of Bacteriology 155:956-65.

46. Oh YK, Kim YJ, Park JY, Lee TH, Kim MS, Park S. 2005. Biohydrogen production from carbon monoxide and water by Rhodopseudomonas palustris P4. Biotechnology and Bioprocess Engineering 10:270-274.

47. Pakpour F, Najafpour G, Tabatabaei M, Tohidfar M, Younesi H. 2014. Biohydrogen production from CO-rich syngas via a locally isolated Rhodopseudomonas palustris PT. Bioprocess and Biosystems Engineering 37:923-930.

48. Lee TH, Park JY, Park S. 2002. Growth of Rhodopseudomonas palustris under phototrophic and non-phototrophic conditions and its CO-dependent H 2 production. Biotechnology Letters 24:91-96.

49. Najafpour, G., Younesi, H., Mohamed, A. R. 2005. Bioconversion of Waste Gases into Biofuel via Fermentation in a Continuous Stirred Tank Bioreactor. Malaysian Journal of Microbiology 1:12-17.

50. Younesi H, Najafpour G, Ku Ismail KS, Mohamed AR, Kamaruddin AH. 2008. Biohydrogen production in a continuous stirred tank bioreactor from synthesis gas by anaerobic photosynthetic bacterium: Rhodopirillum rubrum. Bioresource Technology 99:2612-2619.

51. RuiYan, JiLun. 2010. Hydrogen metabolic pathways of Rhodospirillum rubrum under artificial illumination. Science Bulletin 55:32-37.

52. Kerby RL, Ludden PWRoberts GP. 1995. Carbon monoxide-dependent growth of Rhodospirillum rubrum. Journal of Bacteriology 177:2241-2244.

53. Singer SW, Hirst MB, Ludden PW. 2006. CO-dependent H 2 evolution by Rhodospirillum rubrum : Role of CODH:CooF complex. Biochimica Et Biophysica Acta 1757:1582-1591.

54. Mérida W, Maness PC, Brown RC, Levin DB. 2004. Enhanced hydrogen production from indirectly heated, gasified biomass, and removal of carbon gas emissions using a novel biological gas reformer. International Journal of Hydrogen Energy 29:283-290.

55. Maness PC, Weaver PF. 2002. Hydrogen production from a carbon-monoxide oxidation pathway in Rubrivivax gelatinosus. International Journal of Hydrogen Energy 27:1407-1411.

56. Jensen A, Kai F. 2005. Isolation and characterization of Sulfurospirillum carboxydovorans sp. nov., a new microaerophilic carbon monoxide oxidizing epsilon Proteobacterium. Antonie van Leeuwenhoek 87:339-53.

57. Lupton FS, Conrad R, Zeikus JG. 1984. CO metabolism of Desulfovibrio vulgaris strain Madison: physiological function in the absence or presence of exogeneous substrates. FEMS Microbiology Letters 23:263-268.

58. Jung G, Jung JY, Park S, Kim J. 1999. A new chemoheterotrophic bacterium catalyzing water-gas shift reaction. Biotechnology Letters 21:869-873.

59. Robaire S. 1961. Biological hydrogen production using Citrobacter amalonaticus Y19 to catalyze the water-gas shift reaction. University of British Columbia.
